# Supplementary material for: Biological characteristics of marine Streptomyces SK3 and optimization of cultivation conditions for production of compounds against Vibiriosis pathogen isolated from cultured white shrimp (Litopenaeus vannamei)
Source: PeerJ. 2024 Sep 24;12:e18053. doi: 10.7717/peerj.18053 (PMC11430173; doi:10.7717/peerj.18053)
Supplement: Supplemental Information 1 [file peerj-12-18053-s001.docx]

**Table ST1** Effect of media types to BSMs production

| **Type of medium** | **Inhibition zone (mm)** |
| --- | --- |
| YMB | 30.08±0.58^a^ |
| TSB | 21.08±0.58^d^ |
| MB | 17.08±0.58^e^ |
| MHB | 0.00±0.00^f^ |
| NB | 0.00±0.00^f^ |
| ISP3 | 25.00±0.00^b^ |
| ISP4 | 16.00±0.00^e^ |
| ISP5 | 23.08±0.58^c^ |
| ISP7 | 20.03±0.03^d^ |

**Note:** The data were presented as mean±SD. Means with a different alphabet in the same column are significantly different (p<0.05).

**Table ST2** Media optimization to potential of BSMs production

| **medium** | **Inhibition zone (mm)** |
| --- | --- |
| YM | 28.00±0.00^b^ |
| YM/2 | 34.00±0.00^a^ |
| YM/3 | 24.08±0.58^c^ |
| YM/4 | 18.08±0.58^e^ |
| YM/5 | 11.65±1.15^f^ |
| YM/6 | 11.33±0.00^f^ |
| ISP3 | 20.65±0.58^d^ |
| ISP3/2 | 26.08±0.58^bc^ |
| ISP3/3 | 13.65±1.15^f^ |
| ISP3/4 | 11.65±1.15^f^ |
| ISP3/5 | 0.00±0.00^g^ |
| ISP3/6 | 0.00±0.00^g^ |

**Note:** The data were presented as mean±SD. Means with a different alphabet in the same column are significantly different (p<0.05).

**Table ST3** Protein secretion to liquid medium

| **Time (Hours)** | **Dry weight of cell (mg/ml)** | **total protein**  **(μg/200 μl)** |
| --- | --- | --- |
| 0 | 0.00±0.00^l^ | 0.00±0.00^n^ |
| 4 | 0.12±0.00^k^ | 0.20±0.01^m^ |
| 8 | 0.15±0.03^k^ | 0.30±0.00^l^ |
| 12 | 0.16±0.03^k^ | 0.40±0.00^k^ |
| 16 | 0.28±0.04^j^ | 0.50±0.00^j^ |
| 20 | 0.47±0.05^i^ | 0.60±0.00^i^ |
| 24 | 1.53±0.02^h^ | 0.73±0.00^h^ |
| 48 | 3.41±0.02^g^ | 1.55±0.01^g^ |
| 72 | 6.47±0.02^d^ | 4.90±0.00^f^ |
| 96 | 9.75±0.01^a^ | 8.36±0.01^a^ |
| 120 | 9.75±0.02^a^ | 8.34±0.01^a^ |
| 144 | 9.74±0.05^a^ | 8.34±0.01^a^ |
| 168 | 9.74±0.01^a^ | 7.11±0.01^b^ |
| 192 | 9.74±0.02^a^ | 6.91±0.02^c^ |
| 216 | 7.68±0.02^b^ | 6.42±0.01^d^ |
| 240 | 7.04±0.02^c^ | 6.36±0.01^e^ |
| 264 | 6.02±0.05^e^ | 6.40±0.01^d^ |
| 288 | 5.21±0.02^f^ | 6.40±0.01^d^ |

**Note:** The data were presented as mean±SD. Means with a different alphabet in the same column are significantly different (p<0.05).

**Table ST4** Effect of initial incubation temperature to BSMs production

| **temperature (Cº)** | **Inhibition zone (mm)** |
| --- | --- |
| 10 | 0.00±0.00^d^ |
| 20 | 16.32±0.59^b^ |
| 30 | 28.00±0.00^a^ |
| 40 | 11.74±0.58^c^ |
| 50 | 11.74±0.58^c^ |

**Note:** The data were presented as mean±SD. Means with a different alphabet in the same column are significantly different (p<0.05).

**Table ST5** Effect of incubation period to BSMs production

| **Incubation period (days)** | **Inhibition zone (mm)** |
| --- | --- |
| 1 | 0.00±0.00^e^ |
| 2 | 0.00±0.00^e^ |
| 3 | 16.32±0.58^d^ |
| 4 | 23.32±0.59^c^ |
| 5 | 28.23±0.58^a^ |
| 6 | 28.23±0.58^a^ |
| 7 | 28.00±1.00^ab^ |
| 8 | 26.00±1.00^b^ |
| 9 | 27.39±0.58^ab^ |
| 10 | 27.38±0.58^ab^ |

**Note:** The data were presented as mean±SD. Means with a different alphabet in the same column are significantly different (p<0.05).

**Table ST6** Effect of agitation on BSMs production of *S. hiroshimensis*

| **Agitation speed (× 10 rpm)** | **Inhibition zone (mm)** |
| --- | --- |
| 0 | 0.00±0.00^e^ |
| 100 | 16.32±0.59^d^ |
| 150 | 20.00±0.00^c^ |
| 200 | 35.32±0.58^a^ |
| 250 | 23.34±0.58^b^ |

**Note:** The data were presented as mean±SD. Means with a different alphabet in the same column are significantly different (p<0.05).

**Table ST7** Effect of initial pH of medium on BSMs production of

*S. hiroshimensis*

| **pH** | **Inhibition zone (mm)** |
| --- | --- |
| 3 | 0.00±0.00^f^ |
| 4 | 0.00±0.00^f^ |
| 5 | 14.57±0.58^e^ |
| 6 | 28.37±0.58^b^ |
| 7 | 33.00±0.00^a^ |
| 8 | 27.33±0.58^b^ |
| 9 | 23.00±0.00^c^ |
| 10 | 18.33±0.58^d^ |
| 11 | 15.67±0.58^e^ |
| 12 | 0.00±0.00^f^ |

**Note:** The data were presented as mean±SD. Means with a different alphabet in the same column are significantly different (p<0.05).

**Table ST8** Effect of carbon sources on BCs production of

*S. hiroshimensis*

| **Carbon sources (1%)** | **Inhibition zone (mm)** |
| --- | --- |
| Glucose | 0.00±0.00^f^ |
| Maltose | 15.00±0.58^e^ |
| Sucrose | 17.67±0.00^d^ |
| Glycerol | 20.33±1.00^c^ |
| Starch | 23.00±0.50^b^ |
| unsupplement | 34.33±0.58^a^ |

**Note:** The data were presented as mean±SD. Means with a different alphabet in the same column are significantly different (p<0.05).

**Table ST9** Effect of nitrogen sources on BCs production of
 *S. hiroshimensis*

| **Nitrogen sources (1%)** | **Inhibition zone (mm)** |
| --- | --- |
| Casein | 22.67±0.00^bc^ |
| Peptone | 21.67±0.51^bc^ |
| Beef extract | 20.67±0.00^c^ |
| Malt extract | 23.33±1.00^b^ |
| Urea | 18.00±0.58^d^ |
| unsupplement | 32.67±0.58^a^ |

**Note:** The data were presented as mean±SD. Means with a different alphabet in the same column are significantly different (p<0.05).

**Table ST10** Effect of trace elements on BCs production of
*S. hiroshimensis*

| **Trace elements (1%)** | **Inhibition zone (mm)** |
| --- | --- |
| FeSO_4_ | 0.00±0.00^c^ |
| MgSO_4_ | 33.67±1.00^a^ |
| CaCO_3_ | 33.67±0.58^a^ |
| K_2_HPO_4_ | 0.00±0.00^c^ |
| KH_2_PO_4_ | 19.00±0.58^b^ |
| KCI | 0.00±0.00^c^ |
| KNO_3_ | 0.00±0.00^c^ |
| unsupplement | 33.67±0.58^a^ |

**Note:** The data were presented as mean±SD. Means with a different alphabet in the same column are significantly different (p<0.05).

**Table ST11 Effect of salt concentration on BSMs production**

| **Salt concentration (%)** | **Inhibition zone (mm)** |
| --- | --- |
| 0.0 | 28.67±0.00^b^ |
| 0.5 | 32.67±0.58^a^ |
| 1.0 | 23.33±1.00^c^ |
| 1.5 | 18.00±0.58^d^ |
| 2.0 | 18.67±0.58^d^ |
| 2.5 | 12.33±0.00^e^ |
| 3.0 | 0.00±0.00^f^ |

**Note:** The data were presented as mean±SD. Means with a different alphabet in the same column are significantly different (p<0.05).
