# Supplementary material for: Biological characteristics of marine Streptomyces SK3 and optimization of cultivation conditions for production of compounds against Vibiriosis pathogen isolated from cultured white shrimp (Litopenaeus vannamei)
Source: PeerJ. 2024 Sep 24;12:e18053. doi: 10.7717/peerj.18053 (PMC11430173; doi:10.7717/peerj.18053)
Supplement: Supplemental Information 4 — Raw data exported from the statistical software SPSS (version 22) was analyzed using one-way ANOVA at a 95% confidence interval (p < 0.05) of carbon sources. [file peerj-12-18053-s004.pdf]

```
ONEWAY Inhibition BY Carbon
  /STATISTICS DESCRIPTIVES EFFECTS
  /MISSING ANALYSIS
  /POSTHOC=DUNCAN LSD ALPHA(0.05).
```

Oneway

Notes

Output Created		27-APR-2024 13:29:53
Comments		
Input	Active Dataset	DataSet0
	Filter	<none>
	Weight	<none>
	Split File	<none>
	N of Rows in Working Data File	12
Missing Value Handling	Definition of Missing	User-defined missing values are treated as missing.
	Cases Used	Statistics for each analysis are based on cases with no missing data for any variable in the analysis.
Syntax		ONEWAY Inhibition BY Carbon /STATISTICS DESCRIPTIVES EFFECTS /MISSING ANALYSIS /POSTHOC=DUNCAN LSD ALPHA(0.05).
Resources	Processor Time	00:00:00.03
	Elapsed Time	00:00:00.05

```
[DataSet0]
```

Descriptives

Inhibition

		N	Mean	Std. Deviation	Std. Error	95% Confidence ... Lower Bound
Glucose		2	.0000	.00000	.00000	.0000
Maltose		2	15.0000	.82024	.58000	7.6304
Sucrose		2	17.6700	.00000	.00000	17.6700
Glycerol		2	20.3300	1.41421	1.00000	7.6238
Starch		2	23.0000	.70711	.50000	16.6469
No supplement		2	34.3300	.82024	.58000	26.9604
Total		12	18.3883	10.71299	3.09257	11.5816
Model	Fixed Effects			.80058	.23111	17.8228
	Random Effects				4.58004	6.6150

Descriptives

Inhibition

		95% Confidence Interval for Mean Upper Bound	Minimum	Maximum	Between-Component Variance
Glucose		.0000	.00	.00	
Maltose		22.3696	14.42	15.58	
Sucrose		17.6700	17.67	17.67	
Glycerol		33.0362	19.33	21.33	
Starch		29.3531	22.50	23.50	
No supplement		41.6996	33.75	34.91	
Total		25.1950	.00	34.91	
Model	Fixed Effects	18.9538			
	Random Effects	30.1617			125.53991

ANOVA

Inhibition

	Sum of Squares	df	Mean Square	F	Sig.
Between Groups	1258.604	5	251.721	392.741	.000
Within Groups	3.846	6	.641		
Total	1262.449	11			

Post Hoc Tests

Multiple Comparisons

Dependent Variable: Inhibition

	(I) Carbon	(J) Carbon	Mean Difference (I-J)	Std. Error	Sig.	95% ...
						Lower Bound
LSD	Glucose	Maltose	-15.00000*	.80058	.000	-16.9590
		Sucrose	-17.67000*	.80058	.000	-19.6290
		Glycerol	-20.33000*	.80058	.000	-22.2890
		Starch	-23.00000*	.80058	.000	-24.9590
		No supplement	-34.33000*	.80058	.000	-36.2890
	Maltose	Glucose	15.00000*	.80058	.000	13.0410
		Sucrose	-2.67000*	.80058	.016	-4.6290
		Glycerol	-5.33000*	.80058	.001	-7.2890
		Starch	-8.00000*	.80058	.000	-9.9590
		No supplement	-19.33000*	.80058	.000	-21.2890
	Sucrose	Glucose	17.67000*	.80058	.000	15.7110
		Maltose	2.67000*	.80058	.016	.7110
		Glycerol	-2.66000*	.80058	.016	-4.6190
		Starch	-5.33000*	.80058	.001	-7.2890
		No supplement	-16.66000*	.80058	.000	-18.6190
	Glycerol	Glucose	20.33000*	.80058	.000	18.3710
		Maltose	5.33000*	.80058	.001	3.3710
		Sucrose	2.66000*	.80058	.016	.7010
		Starch	-2.67000*	.80058	.016	-4.6290
		No supplement	-14.00000*	.80058	.000	-15.9590
	Starch	Glucose	23.00000*	.80058	.000	21.0410
		Maltose	8.00000*	.80058	.000	6.0410
		Sucrose	5.33000*	.80058	.001	3.3710
		Glycerol	2.67000*	.80058	.016	.7110
		No supplement	-11.33000*	.80058	.000	-13.2890
	No supplement	Glucose	34.33000*	.80058	.000	32.3710
		Maltose	19.33000*	.80058	.000	17.3710
		Sucrose	16.66000*	.80058	.000	14.7010
		Glycerol	14.00000*	.80058	.000	12.0410
		Starch	11.33000*	.80058	.000	9.3710

Multiple Comparisons

Dependent Variable: Inhibition

	(I) Carbon	(J) Carbon	95% Confidence Upper Bound
LSD	Glucose	Maltose	-13.0410
		Sucrose	-15.7110
		Glycerol	-18.3710
		Starch	-21.0410
		No supplement	-32.3710
	Maltose	Glucose	16.9590
		Sucrose	-.7110
		Glycerol	-3.3710
		Starch	-6.0410
		No supplement	-17.3710
	Sucrose	Glucose	19.6290
		Maltose	4.6290
		Glycerol	-.7010
		Starch	-3.3710
		No supplement	-14.7010
	Glycerol	Glucose	22.2890
		Maltose	7.2890
		Sucrose	4.6190
		Starch	-.7110
		No supplement	-12.0410
	Starch	Glucose	24.9590
		Maltose	9.9590
		Sucrose	7.2890
		Glycerol	4.6290
		No supplement	-9.3710
	No supplement	Glucose	36.2890
		Maltose	21.2890
		Sucrose	18.6190
		Glycerol	15.9590
		Starch	13.2890

*. The mean difference is significant at the 0.05 level.

Homogeneous Subsets

Inhibition

	Carbon	N	Subset for alpha = 0.05				
			1	2	3	4	5
Duncan[a]	Glucose	2	.0000				
	Maltose	2		15.0000			
	Sucrose	2			17.6700		
	Glycerol	2				20.3300	
	Starch	2					23.0000
	No supplement	2					
	Sig.		1.000	1.000	1.000	1.000	1.000

Inhibition

	Carbon	Subset for .
		6
Duncan[a]	Glucose	
	Maltose	
	Sucrose	
	Glycerol	
	Starch	
	No supplement	34.3300
	Sig.	1.000

Means for groups in homogeneous subsets are displayed.

a. Uses Harmonic Mean Sample Size = 2.000.
