# Supplementary material for: Biological characteristics of marine Streptomyces SK3 and optimization of cultivation conditions for production of compounds against Vibiriosis pathogen isolated from cultured white shrimp (Litopenaeus vannamei)
Source: PeerJ. 2024 Sep 24;12:e18053. doi: 10.7717/peerj.18053 (PMC11430173; doi:10.7717/peerj.18053)
Supplement: Supplemental Information 5 — Raw data exported from the statistical software SPSS (version 22) was analyzed using one-way ANOVA at a 95% confidence interval (p < 0.05) of protein secretion to liquid medium. [file peerj-12-18053-s005.pdf]

```
ONEWAY weight BY Time
  /STATISTICS DESCRIPTIVES EFFECTS
  /MISSING ANALYSIS
  /POSTHOC=DUNCAN LSD ALPHA(0.05).
```

Oneway

Notes

Output Created		27-APR-2024 11:57:32
Comments		
Input	Active Dataset	DataSet0
	Filter	<none>
	Weight	<none>
	Split File	<none>
	N of Rows in Working Data File	36
Missing Value Handling	Definition of Missing	User-defined missing values are treated as missing.
	Cases Used	Statistics for each analysis are based on cases with no missing data for any variable in the analysis.
Syntax		ONEWAY weight BY Time /STATISTICS DESCRIPTIVES EFFECTS /MISSING ANALYSIS /POSTHOC=DUNCAN LSD ALPHA(0.05).
Resources	Processor Time	00:00:00.14
	Elapsed Time	00:00:00.33

Descriptives

weight

		N	Mean	Std. Deviation	Std. Error	95% Confidence ...
						Lower Bound
0		2	.00000	.000000	.000000	.00000
4		2	.12050	.000707	.000500	.11415
8		2	.15150	.041719	.029500	-.22333
12		2	.16400	.035355	.025000	-.15366
16		2	.28200	.059397	.042000	-.25166
20		2	.47000	.070711	.050000	-.16531
24		2	1.52900	.028284	.020000	1.27488
48		2	3.41300	.031113	.022000	3.13346
72		2	6.46700	.021213	.015000	6.27641
96		2	9.75350	.014849	.010500	9.62008
120		2	9.74600	.031113	.022000	9.46646
144		2	9.74000	.070711	.050000	9.10469
168		2	9.74450	.013435	.009500	9.62379
192		2	9.74400	.021213	.015000	9.55341
216		2	7.68100	.028284	.020000	7.42688
240		2	7.04100	.028284	.020000	6.78688
264		2	6.02000	.070711	.050000	5.38469
288		2	5.20550	.028991	.020500	4.94502
Total		36	4.84847	3.998884	.666481	3.49544
Model	Fixed Effects			.039438	.006573	4.83466
	Random Effects				.956283	2.83089

Descriptives

weight

		95% Confidence Interval for Mean			
		Upper Bound	Minimum	Maximum	Between-Component Variance
0		.00000	.000	.000	
4		.12685	.120	.121	
8		.52633	.122	.181	
12		.48166	.139	.189	
16		.81566	.240	.324	
20		1.10531	.420	.520	
24		1.78312	1.509	1.549	
48		3.69254	3.391	3.435	
72		6.65759	6.452	6.482	
96		9.88692	9.743	9.764	
120		10.02554	9.724	9.768	
144		10.37531	9.690	9.790	
168		9.86521	9.735	9.754	
192		9.93459	9.729	9.759	
216		7.93512	7.661	7.701	
240		7.29512	7.021	7.061	
264		6.65531	5.970	6.070	
288		5.46598	5.185	5.226	
Total		6.20150	.000	9.790	
Model	Fixed Effects	4.86228			
	Random Effects	6.86605			16.459797

ANOVA

weight

	Sum of Squares	df	Mean Square	F	Sig.
Between Groups	559.660	17	32.921	21166.242	.000
Within Groups	.028	18	.002		
Total	559.688	35			

Post Hoc Tests

Multiple Comparisons

Dependent Variable: weight

	(I) Time	(J) Time	Mean Difference (I-J)	Std. Error	Sig.	95% Confidence Interval	
						Lower Bound	Upper Bound
LSD	0	4	-.120500*	.039438	.007	-.20336	-.03764
		8	-.151500*	.039438	.001	-.23436	-.06864
		12	-.164000*	.039438	.001	-.24686	-.08114
		16	-.282000*	.039438	.000	-.36486	-.19914
		20	-.470000*	.039438	.000	-.55286	-.38714
		24	-1.529000*	.039438	.000	-1.61186	-1.44614
		48	-3.413000*	.039438	.000	-3.49586	-3.33014
		72	-6.467000*	.039438	.000	-6.54986	-6.38414
		96	-9.753500*	.039438	.000	-9.83636	-9.67064
		120	-9.746000*	.039438	.000	-9.82886	-9.66314
		144	-9.740000*	.039438	.000	-9.82286	-9.65714
		168	-9.744500*	.039438	.000	-9.82736	-9.66164
		192	-9.744000*	.039438	.000	-9.82686	-9.66114
		216	-7.681000*	.039438	.000	-7.76386	-7.59814
		240	-7.041000*	.039438	.000	-7.12386	-6.95814
		264	-6.020000*	.039438	.000	-6.10286	-5.93714
		288	-5.205500*	.039438	.000	-5.28836	-5.12264
	4	0	.120500*	.039438	.007	.03764	.20336
		8	-.031000	.039438	.442	-.11386	.05186
		12	-.043500	.039438	.285	-.12636	.03936
		16	-.161500*	.039438	.001	-.24436	-.07864
		20	-.349500*	.039438	.000	-.43236	-.26664
		24	-1.408500*	.039438	.000	-1.49136	-1.32564
		48	-3.292500*	.039438	.000	-3.37536	-3.20964
		72	-6.346500*	.039438	.000	-6.42936	-6.26364
		96	-9.633000*	.039438	.000	-9.71586	-9.55014
		120	-9.625500*	.039438	.000	-9.70836	-9.54264
		144	-9.619500*	.039438	.000	-9.70236	-9.53664
		168	-9.624000*	.039438	.000	-9.70686	-9.54114
		192	-9.623500*	.039438	.000	-9.70636	-9.54064
		216	-7.560500*	.039438	.000	-7.64336	-7.47764
		240	-6.920500*	.039438	.000	-7.00336	-6.83764
		264	-5.899500*	.039438	.000	-5.98236	-5.81664
		288	-5.085000*	.039438	.000	-5.16786	-5.00214
	8	0	.151500*	.039438	.001	.06864	.23436
		4	.031000	.039438	.442	-.05186	.11386
		12	-.012500	.039438	.755	-.09536	.07036

Multiple Comparisons

Dependent Variable: weight

(I) Time	(J) Time	Mean Difference (I-J)	Std. Error	Sig.	95% Confidence Interval Lower Bound	Upper Bound
	16	-.130500*	.039438	.004	-.21336	-.04764
	20	-.318500*	.039438	.000	-.40136	-.23564
	24	-1.377500*	.039438	.000	-1.46036	-1.29464
	48	-3.261500*	.039438	.000	-3.34436	-3.17864
	72	-6.315500*	.039438	.000	-6.39836	-6.23264
	96	-9.602000*	.039438	.000	-9.68486	-9.51914
	120	-9.594500*	.039438	.000	-9.67736	-9.51164
	144	-9.588500*	.039438	.000	-9.67136	-9.50564
	168	-9.593000*	.039438	.000	-9.67586	-9.51014
	192	-9.592500*	.039438	.000	-9.67536	-9.50964
	216	-7.529500*	.039438	.000	-7.61236	-7.44664
	240	-6.889500*	.039438	.000	-6.97236	-6.80664
	264	-5.868500*	.039438	.000	-5.95136	-5.78564
	288	-5.054000*	.039438	.000	-5.13686	-4.97114
12	0	.164000*	.039438	.001	.08114	.24686
	4	.043500	.039438	.285	-.03936	.12636
	8	.012500	.039438	.755	-.07036	.09536
	16	-.118000*	.039438	.008	-.20086	-.03514
	20	-.306000*	.039438	.000	-.38886	-.22314
	24	-1.365000*	.039438	.000	-1.44786	-1.28214
	48	-3.249000*	.039438	.000	-3.33186	-3.16614
	72	-6.303000*	.039438	.000	-6.38586	-6.22014
	96	-9.589500*	.039438	.000	-9.67236	-9.50664
	120	-9.582000*	.039438	.000	-9.66486	-9.49914
	144	-9.576000*	.039438	.000	-9.65886	-9.49314
	168	-9.580500*	.039438	.000	-9.66336	-9.49764
	192	-9.580000*	.039438	.000	-9.66286	-9.49714
	216	-7.517000*	.039438	.000	-7.59986	-7.43414
	240	-6.877000*	.039438	.000	-6.95986	-6.79414
	264	-5.856000*	.039438	.000	-5.93886	-5.77314
	288	-5.041500*	.039438	.000	-5.12436	-4.95864
16	0	.282000*	.039438	.000	.19914	.36486
	4	.161500*	.039438	.001	.07864	.24436
	8	.130500*	.039438	.004	.04764	.21336
	12	.118000*	.039438	.008	.03514	.20086
	20	-.188000*	.039438	.000	-.27086	-.10514
	24	-1.247000*	.039438	.000	-1.32986	-1.16414

Multiple Comparisons

Dependent Variable: weight

(I) Time	(J) Time	Mean Difference (I-J)	Std. Error	Sig.	95% Confidence Interval Lower Bound	Upper Bound
	48	-3.131000*	.039438	.000	-3.21386	-3.04814
	72	-6.185000*	.039438	.000	-6.26786	-6.10214
	96	-9.471500*	.039438	.000	-9.55436	-9.38864
	120	-9.464000*	.039438	.000	-9.54686	-9.38114
	144	-9.458000*	.039438	.000	-9.54086	-9.37514
	168	-9.462500*	.039438	.000	-9.54536	-9.37964
	192	-9.462000*	.039438	.000	-9.54486	-9.37914
	216	-7.399000*	.039438	.000	-7.48186	-7.31614
	240	-6.759000*	.039438	.000	-6.84186	-6.67614
	264	-5.738000*	.039438	.000	-5.82086	-5.65514
	288	-4.923500*	.039438	.000	-5.00636	-4.84064
20	0	.470000*	.039438	.000	.38714	.55286
	4	.349500*	.039438	.000	.26664	.43236
	8	.318500*	.039438	.000	.23564	.40136
	12	.306000*	.039438	.000	.22314	.38886
	16	.188000*	.039438	.000	.10514	.27086
	24	-1.059000*	.039438	.000	-1.14186	-.97614
	48	-2.943000*	.039438	.000	-3.02586	-2.86014
	72	-5.997000*	.039438	.000	-6.07986	-5.91414
	96	-9.283500*	.039438	.000	-9.36636	-9.20064
	120	-9.276000*	.039438	.000	-9.35886	-9.19314
	144	-9.270000*	.039438	.000	-9.35286	-9.18714
	168	-9.274500*	.039438	.000	-9.35736	-9.19164
	192	-9.274000*	.039438	.000	-9.35686	-9.19114
	216	-7.211000*	.039438	.000	-7.29386	-7.12814
	240	-6.571000*	.039438	.000	-6.65386	-6.48814
	264	-5.550000*	.039438	.000	-5.63286	-5.46714
	288	-4.735500*	.039438	.000	-4.81836	-4.65264
24	0	1.529000*	.039438	.000	1.44614	1.61186
	4	1.408500*	.039438	.000	1.32564	1.49136
	8	1.377500*	.039438	.000	1.29464	1.46036
	12	1.365000*	.039438	.000	1.28214	1.44786
	16	1.247000*	.039438	.000	1.16414	1.32986
	20	1.059000*	.039438	.000	.97614	1.14186
	48	-1.884000*	.039438	.000	-1.96686	-1.80114
	72	-4.938000*	.039438	.000	-5.02086	-4.85514
	96	-8.224500*	.039438	.000	-8.30736	-8.14164

Multiple Comparisons

Dependent Variable: weight

(I) Time	(J) Time	Mean Difference (I-J)	Std. Error	Sig.	95% Confidence Interval Lower Bound	Upper Bound
	120	-8.217000*	.039438	.000	-8.29986	-8.13414
	144	-8.211000*	.039438	.000	-8.29386	-8.12814
	168	-8.215500*	.039438	.000	-8.29836	-8.13264
	192	-8.215000*	.039438	.000	-8.29786	-8.13214
	216	-6.152000*	.039438	.000	-6.23486	-6.06914
	240	-5.512000*	.039438	.000	-5.59486	-5.42914
	264	-4.491000*	.039438	.000	-4.57386	-4.40814
	288	-3.676500*	.039438	.000	-3.75936	-3.59364
48	0	3.413000*	.039438	.000	3.33014	3.49586
	4	3.292500*	.039438	.000	3.20964	3.37536
	8	3.261500*	.039438	.000	3.17864	3.34436
	12	3.249000*	.039438	.000	3.16614	3.33186
	16	3.131000*	.039438	.000	3.04814	3.21386
	20	2.943000*	.039438	.000	2.86014	3.02586
	24	1.884000*	.039438	.000	1.80114	1.96686
	72	-3.054000*	.039438	.000	-3.13686	-2.97114
	96	-6.340500*	.039438	.000	-6.42336	-6.25764
	120	-6.333000*	.039438	.000	-6.41586	-6.25014
	144	-6.327000*	.039438	.000	-6.40986	-6.24414
	168	-6.331500*	.039438	.000	-6.41436	-6.24864
	192	-6.331000*	.039438	.000	-6.41386	-6.24814
	216	-4.268000*	.039438	.000	-4.35086	-4.18514
	240	-3.628000*	.039438	.000	-3.71086	-3.54514
	264	-2.607000*	.039438	.000	-2.68986	-2.52414
	288	-1.792500*	.039438	.000	-1.87536	-1.70964
72	0	6.467000*	.039438	.000	6.38414	6.54986
	4	6.346500*	.039438	.000	6.26364	6.42936
	8	6.315500*	.039438	.000	6.23264	6.39836
	12	6.303000*	.039438	.000	6.22014	6.38586
	16	6.185000*	.039438	.000	6.10214	6.26786
	20	5.997000*	.039438	.000	5.91414	6.07986
	24	4.938000*	.039438	.000	4.85514	5.02086
	48	3.054000*	.039438	.000	2.97114	3.13686
	96	-3.286500*	.039438	.000	-3.36936	-3.20364
	120	-3.279000*	.039438	.000	-3.36186	-3.19614
	144	-3.273000*	.039438	.000	-3.35586	-3.19014
	168	-3.277500*	.039438	.000	-3.36036	-3.19464

Multiple Comparisons

Dependent Variable: weight

(I) Time	(J) Time	Mean Difference (I-J)	Std. Error	Sig.	95% Confidence Interval Lower Bound	Upper Bound
	192	-3.277000*	.039438	.000	-3.35986	-3.19414
	216	-1.214000*	.039438	.000	-1.29686	-1.13114
	240	-.574000*	.039438	.000	-.65686	-.49114
	264	.447000*	.039438	.000	.36414	.52986
	288	1.261500*	.039438	.000	1.17864	1.34436
96	0	9.753500*	.039438	.000	9.67064	9.83636
	4	9.633000*	.039438	.000	9.55014	9.71586
	8	9.602000*	.039438	.000	9.51914	9.68486
	12	9.589500*	.039438	.000	9.50664	9.67236
	16	9.471500*	.039438	.000	9.38864	9.55436
	20	9.283500*	.039438	.000	9.20064	9.36636
	24	8.224500*	.039438	.000	8.14164	8.30736
	48	6.340500*	.039438	.000	6.25764	6.42336
	72	3.286500*	.039438	.000	3.20364	3.36936
	120	.007500	.039438	.851	-.07536	.09036
	144	.013500	.039438	.736	-.06936	.09636
	168	.009000	.039438	.822	-.07386	.09186
	192	.009500	.039438	.812	-.07336	.09236
	216	2.072500*	.039438	.000	1.98964	2.15536
	240	2.712500*	.039438	.000	2.62964	2.79536
	264	3.733500*	.039438	.000	3.65064	3.81636
	288	4.548000*	.039438	.000	4.46514	4.63086
120	0	9.746000*	.039438	.000	9.66314	9.82886
	4	9.625500*	.039438	.000	9.54264	9.70836
	8	9.594500*	.039438	.000	9.51164	9.67736
	12	9.582000*	.039438	.000	9.49914	9.66486
	16	9.464000*	.039438	.000	9.38114	9.54686
	20	9.276000*	.039438	.000	9.19314	9.35886
	24	8.217000*	.039438	.000	8.13414	8.29986
	48	6.333000*	.039438	.000	6.25014	6.41586
	72	3.279000*	.039438	.000	3.19614	3.36186
	96	-.007500	.039438	.851	-.09036	.07536
	144	.006000	.039438	.881	-.07686	.08886
	168	.001500	.039438	.970	-.08136	.08436
	192	.002000	.039438	.960	-.08086	.08486
	216	2.065000*	.039438	.000	1.98214	2.14786
	240	2.705000*	.039438	.000	2.62214	2.78786

Multiple Comparisons

Dependent Variable: weight

(I) Time	(J) Time	Mean Difference (I-J)	Std. Error	Sig.	95% Confidence Interval Lower Bound	95% Confidence Interval Upper Bound
	264	3.726000*	.039438	.000	3.64314	3.80886
	288	4.540500*	.039438	.000	4.45764	4.62336
144	0	9.740000*	.039438	.000	9.65714	9.82286
	4	9.619500*	.039438	.000	9.53664	9.70236
	8	9.588500*	.039438	.000	9.50564	9.67136
	12	9.576000*	.039438	.000	9.49314	9.65886
	16	9.458000*	.039438	.000	9.37514	9.54086
	20	9.270000*	.039438	.000	9.18714	9.35286
	24	8.211000*	.039438	.000	8.12814	8.29386
	48	6.327000*	.039438	.000	6.24414	6.40986
	72	3.273000*	.039438	.000	3.19014	3.35586
	96	-.013500	.039438	.736	-.09636	.06936
	120	-.006000	.039438	.881	-.08886	.07686
	168	-.004500	.039438	.910	-.08736	.07836
	192	-.004000	.039438	.920	-.08686	.07886
	216	2.059000*	.039438	.000	1.97614	2.14186
	240	2.699000*	.039438	.000	2.61614	2.78186
	264	3.720000*	.039438	.000	3.63714	3.80286
	288	4.534500*	.039438	.000	4.45164	4.61736
168	0	9.744500*	.039438	.000	9.66164	9.82736
	4	9.624000*	.039438	.000	9.54114	9.70686
	8	9.593000*	.039438	.000	9.51014	9.67586
	12	9.580500*	.039438	.000	9.49764	9.66336
	16	9.462500*	.039438	.000	9.37964	9.54536
	20	9.274500*	.039438	.000	9.19164	9.35736
	24	8.215500*	.039438	.000	8.13264	8.29836
	48	6.331500*	.039438	.000	6.24864	6.41436
	72	3.277500*	.039438	.000	3.19464	3.36036
	96	-.009000	.039438	.822	-.09186	.07386
	120	-.001500	.039438	.970	-.08436	.08136
	144	.004500	.039438	.910	-.07836	.08736
	192	.000500	.039438	.990	-.08236	.08336
	216	2.063500*	.039438	.000	1.98064	2.14636
	240	2.703500*	.039438	.000	2.62064	2.78636
	264	3.724500*	.039438	.000	3.64164	3.80736
	288	4.539000*	.039438	.000	4.45614	4.62186

Multiple Comparisons

Dependent Variable: weight

(I) Time	(J) Time	Mean Difference (I-J)	Std. Error	Sig.	95% Confidence Interval Lower Bound	Upper Bound
192	0	9.744000*	.039438	.000	9.66114	9.82686
	4	9.623500*	.039438	.000	9.54064	9.70636
	8	9.592500*	.039438	.000	9.50964	9.67536
	12	9.580000*	.039438	.000	9.49714	9.66286
	16	9.462000*	.039438	.000	9.37914	9.54486
	20	9.274000*	.039438	.000	9.19114	9.35686
	24	8.215000*	.039438	.000	8.13214	8.29786
	48	6.331000*	.039438	.000	6.24814	6.41386
	72	3.277000*	.039438	.000	3.19414	3.35986
	96	-.009500	.039438	.812	-.09236	.07336
	120	-.002000	.039438	.960	-.08486	.08086
	144	.004000	.039438	.920	-.07886	.08686
	168	-.000500	.039438	.990	-.08336	.08236
	216	2.063000*	.039438	.000	1.98014	2.14586
	240	2.703000*	.039438	.000	2.62014	2.78586
	264	3.724000*	.039438	.000	3.64114	3.80686
	288	4.538500*	.039438	.000	4.45564	4.62136
216	0	7.681000*	.039438	.000	7.59814	7.76386
	4	7.560500*	.039438	.000	7.47764	7.64336
	8	7.529500*	.039438	.000	7.44664	7.61236
	12	7.517000*	.039438	.000	7.43414	7.59986
	16	7.399000*	.039438	.000	7.31614	7.48186
	20	7.211000*	.039438	.000	7.12814	7.29386
	24	6.152000*	.039438	.000	6.06914	6.23486
	48	4.268000*	.039438	.000	4.18514	4.35086
	72	1.214000*	.039438	.000	1.13114	1.29686
	96	-2.072500*	.039438	.000	-2.15536	-1.98964
	120	-2.065000*	.039438	.000	-2.14786	-1.98214
	144	-2.059000*	.039438	.000	-2.14186	-1.97614
	168	-2.063500*	.039438	.000	-2.14636	-1.98064
	192	-2.063000*	.039438	.000	-2.14586	-1.98014
	240	.640000*	.039438	.000	.55714	.72286
	264	1.661000*	.039438	.000	1.57814	1.74386
	288	2.475500*	.039438	.000	2.39264	2.55836
240	0	7.041000*	.039438	.000	6.95814	7.12386
	4	6.920500*	.039438	.000	6.83764	7.00336
	8	6.889500*	.039438	.000	6.80664	6.97236

Multiple Comparisons

Dependent Variable: weight

(I) Time	(J) Time	Mean Difference (I-J)	Std. Error	Sig.	95% Confidence Interval Lower Bound	95% Confidence Interval Upper Bound
	12	6.877000*	.039438	.000	6.79414	6.95986
	16	6.759000*	.039438	.000	6.67614	6.84186
	20	6.571000*	.039438	.000	6.48814	6.65386
	24	5.512000*	.039438	.000	5.42914	5.59486
	48	3.628000*	.039438	.000	3.54514	3.71086
	72	.574000*	.039438	.000	.49114	.65686
	96	-2.712500*	.039438	.000	-2.79536	-2.62964
	120	-2.705000*	.039438	.000	-2.78786	-2.62214
	144	-2.699000*	.039438	.000	-2.78186	-2.61614
	168	-2.703500*	.039438	.000	-2.78636	-2.62064
	192	-2.703000*	.039438	.000	-2.78586	-2.62014
	216	-.640000*	.039438	.000	-.72286	-.55714
	264	1.021000*	.039438	.000	.93814	1.10386
	288	1.835500*	.039438	.000	1.75264	1.91836
264	0	6.020000*	.039438	.000	5.93714	6.10286
	4	5.899500*	.039438	.000	5.81664	5.98236
	8	5.868500*	.039438	.000	5.78564	5.95136
	12	5.856000*	.039438	.000	5.77314	5.93886
	16	5.738000*	.039438	.000	5.65514	5.82086
	20	5.550000*	.039438	.000	5.46714	5.63286
	24	4.491000*	.039438	.000	4.40814	4.57386
	48	2.607000*	.039438	.000	2.52414	2.68986
	72	-.447000*	.039438	.000	-.52986	-.36414
	96	-3.733500*	.039438	.000	-3.81636	-3.65064
	120	-3.726000*	.039438	.000	-3.80886	-3.64314
	144	-3.720000*	.039438	.000	-3.80286	-3.63714
	168	-3.724500*	.039438	.000	-3.80736	-3.64164
	192	-3.724000*	.039438	.000	-3.80686	-3.64114
	216	-1.661000*	.039438	.000	-1.74386	-1.57814
	240	-1.021000*	.039438	.000	-1.10386	-.93814
	288	.814500*	.039438	.000	.73164	.89736
288	0	5.205500*	.039438	.000	5.12264	5.28836
	4	5.085000*	.039438	.000	5.00214	5.16786
	8	5.054000*	.039438	.000	4.97114	5.13686
	12	5.041500*	.039438	.000	4.95864	5.12436
	16	4.923500*	.039438	.000	4.84064	5.00636
	20	4.735500*	.039438	.000	4.65264	4.81836

Multiple Comparisons

Dependent Variable: weight

(I) Time	(J) Time	Mean Difference (I-J)	Std. Error	Sig.	95% Confidence Interval	
					Lower Bound	Upper Bound
	24	3.676500*	.039438	.000	3.59364	3.75936
	48	1.792500*	.039438	.000	1.70964	1.87536
	72	-1.261500*	.039438	.000	-1.34436	-1.17864
	96	-4.548000*	.039438	.000	-4.63086	-4.46514
	120	-4.540500*	.039438	.000	-4.62336	-4.45764
	144	-4.534500*	.039438	.000	-4.61736	-4.45164
	168	-4.539000*	.039438	.000	-4.62186	-4.45614
	192	-4.538500*	.039438	.000	-4.62136	-4.45564
	216	-2.475500*	.039438	.000	-2.55836	-2.39264
	240	-1.835500*	.039438	.000	-1.91836	-1.75264
	264	-.814500*	.039438	.000	-.89736	-.73164

*. The mean difference is significant at the 0.05 level.

Homogeneous Subsets

weight

	Time	N	Subset for alpha = 0.05					
			1	2	3	4	5	6
Duncan[a]	0	2	.00000					
	4	2		.12050				
	8	2		.15150				
	12	2		.16400				
	16	2			.28200			
	20	2				.47000		
	24	2					1.52900	
	48	2						3.41300
	288	2						
	264	2						
	72	2						
	240	2						
	216	2						
	144	2						
	192	2						
	168	2						
	120	2						
	96	2						
	Sig.		1.000	.310	1.000	1.000	1.000	1.000

weight

	Time	Subset for alpha = 0.05					
		7	8	9	10	11	12
Duncan[a]	0						
	4						
	8						
	12						
	16						
	20						
	24						
	48						
	288	5.20550					
	264		6.02000				
	72			6.46700			
	240				7.04100		
	216					7.68100	
	144						9.74000
	192						9.74400
	168						9.74450
	120						9.74600
	96						9.75350
	Sig.	1.000	1.000	1.000	1.000	1.000	.762

Means for groups in homogeneous subsets are displayed.

a. Uses Harmonic Mean Sample Size = 2.000.
