# Supplementary material for: Biological characteristics of marine Streptomyces SK3 and optimization of cultivation conditions for production of compounds against Vibiriosis pathogen isolated from cultured white shrimp (Litopenaeus vannamei)
Source: PeerJ. 2024 Sep 24;12:e18053. doi: 10.7717/peerj.18053 (PMC11430173; doi:10.7717/peerj.18053)
Supplement: Supplemental Information 6 — Raw data exported from the statistical software SPSS (version 22) was analyzed using one-way ANOVA at a 95% confidence interval (p < 0.05) of incubation period. [file peerj-12-18053-s006.pdf]

```
ONEWAY Inhibition BY Incubation
  /STATISTICS DESCRIPTIVES EFFECTS
  /MISSING ANALYSIS
  /POSTHOC=DUNCAN LSD ALPHA(0.05).
```

Oneway

Notes

Output Created		27-APR-2024 12:42:00
Comments		
Input	Active Dataset	DataSet0
	Filter	<none>
	Weight	<none>
	Split File	<none>
	N of Rows in Working Data File	21
Missing Value Handling	Definition of Missing	User-defined missing values are treated as missing.
	Cases Used	Statistics for each analysis are based on cases with no missing data for any variable in the analysis.
Syntax		ONEWAY Inhibition BY Incubation<br>/STATISTICS DESCRIPTIVES EFFECTS<br>/MISSING ANALYSIS<br>/POSTHOC=DUNCAN LSD ALPHA(0.05).
Resources	Processor Time	00:00:00.03
	Elapsed Time	00:00:00.10

```
[DataSet0]
```

Descriptives

Inhibition

		N	Mean	Std. Deviation	Std. Error	95% Confidence ...
						Lower Bound
day1		2	.0000	.00000	.00000	.0000
day2		2	.0000	.00000	.00000	.0000
day3		2	16.3200	.82024	.58000	8.9504
day4		2	23.3150	.82731	.58500	15.8819
day5		2	28.2250	.81317	.57500	20.9189
day6		2	28.2300	.82024	.58000	20.8604
day7		2	28.0000	1.41421	1.00000	15.2938
day8		2	26.0000	1.41421	1.00000	13.2938
day9		2	27.3900	.82024	.58000	20.0204
day10		2	27.3800	.82024	.58000	20.0104
Total		20	20.4860	11.10073	2.48220	15.2907
Model	Fixed Effects			.89649	.20046	20.0393
	Random Effects				3.60035	12.3414

Descriptives

Inhibition

		95% Confidence Interval for Mean	Minimum	Maximum	Between-Component Variance
		Upper Bound			
day1		.0000	.00	.00	
day2		.0000	.00	.00	
day3		23.6896	15.74	16.90	
day4		30.7481	22.73	23.90	
day5		35.5311	27.65	28.80	
day6		35.5996	27.65	28.81	
day7		40.7062	27.00	29.00	
day8		38.7062	25.00	27.00	
day9		34.7596	26.81	27.97	
day10		34.7496	26.80	27.96	
Total		25.6813	.00	29.00	
Model	Fixed Effects	20.9327			
	Random Effects	28.6306			129.22368

ANOVA

Inhibition

	Sum of Squares	df	Mean Square	F	Sig.
Between Groups	2333.259	9	259.251	322.576	.000
Within Groups	8.037	10	.804		
Total	2341.296	19			

Post Hoc Tests

Multiple Comparisons

Dependent Variable: Inhibition

	(I) Incubation	(J) Incubation	Mean Difference (I-J)	Std. Error	Sig.	95% ... Lower Bound
LSD	day1	day2	.00000	.89649	1.000	-1.9975
		day3	-16.32000*	.89649	.000	-18.3175
		day4	-23.31500*	.89649	.000	-25.3125
		day5	-28.22500*	.89649	.000	-30.2225
		day6	-28.23000*	.89649	.000	-30.2275
		day7	-28.00000*	.89649	.000	-29.9975
		day8	-26.00000*	.89649	.000	-27.9975
		day9	-27.39000*	.89649	.000	-29.3875
		day10	-27.38000*	.89649	.000	-29.3775
	day2	day1	.00000	.89649	1.000	-1.9975
		day3	-16.32000*	.89649	.000	-18.3175
		day4	-23.31500*	.89649	.000	-25.3125
		day5	-28.22500*	.89649	.000	-30.2225
		day6	-28.23000*	.89649	.000	-30.2275
		day7	-28.00000*	.89649	.000	-29.9975
		day8	-26.00000*	.89649	.000	-27.9975
		day9	-27.39000*	.89649	.000	-29.3875
		day10	-27.38000*	.89649	.000	-29.3775
	day3	day1	16.32000*	.89649	.000	14.3225
		day2	16.32000*	.89649	.000	14.3225
		day4	-6.99500*	.89649	.000	-8.9925
		day5	-11.90500*	.89649	.000	-13.9025
		day6	-11.91000*	.89649	.000	-13.9075
		day7	-11.68000*	.89649	.000	-13.6775
		day8	-9.68000*	.89649	.000	-11.6775
		day9	-11.07000*	.89649	.000	-13.0675
		day10	-11.06000*	.89649	.000	-13.0575

Multiple Comparisons

Dependent Variable: Inhibition

	(I) Incubation	(J) Incubation	95% Confidence Upper Bound
LSD	day1	day2	1.9975
		day3	-14.3225
		day4	-21.3175
		day5	-26.2275
		day6	-26.2325
		day7	-26.0025
		day8	-24.0025
		day9	-25.3925
		day10	-25.3825
	day2	day1	1.9975
		day3	-14.3225
		day4	-21.3175
		day5	-26.2275
		day6	-26.2325
		day7	-26.0025
		day8	-24.0025
		day9	-25.3925
		day10	-25.3825
	day3	day1	18.3175
		day2	18.3175
		day4	-4.9975
		day5	-9.9075
		day6	-9.9125
		day7	-9.6825
		day8	-7.6825
		day9	-9.0725
		day10	-9.0625

Multiple Comparisons

Dependent Variable: Inhibition

(I) Incubation	(J) Incubation	Mean Difference (I-J)	Std. Error	Sig.	95% ... Lower Bound
day4	day1	23.31500*	.89649	.000	21.3175
	day2	23.31500*	.89649	.000	21.3175
	day3	6.99500*	.89649	.000	4.9975
	day5	-4.91000*	.89649	.000	-6.9075
	day6	-4.91500*	.89649	.000	-6.9125
	day7	-4.68500*	.89649	.000	-6.6825
	day8	-2.68500*	.89649	.013	-4.6825
	day9	-4.07500*	.89649	.001	-6.0725
	day10	-4.06500*	.89649	.001	-6.0625
day5	day1	28.22500*	.89649	.000	26.2275
	day2	28.22500*	.89649	.000	26.2275
	day3	11.90500*	.89649	.000	9.9075
	day4	4.91000*	.89649	.000	2.9125
	day6	-.00500	.89649	.996	-2.0025
	day7	.22500	.89649	.807	-1.7725
	day8	2.22500*	.89649	.032	.2275
	day9	.83500	.89649	.374	-1.1625
	day10	.84500	.89649	.368	-1.1525
day6	day1	28.23000*	.89649	.000	26.2325
	day2	28.23000*	.89649	.000	26.2325
	day3	11.91000*	.89649	.000	9.9125
	day4	4.91500*	.89649	.000	2.9175
	day5	.00500	.89649	.996	-1.9925
	day7	.23000	.89649	.803	-1.7675
	day8	2.23000*	.89649	.032	.2325
	day9	.84000	.89649	.371	-1.1575
	day10	.85000	.89649	.365	-1.1475
day7	day1	28.00000*	.89649	.000	26.0025
	day2	28.00000*	.89649	.000	26.0025
	day3	11.68000*	.89649	.000	9.6825
	day4	4.68500*	.89649	.000	2.6875
	day5	-.22500	.89649	.807	-2.2225
	day6	-.23000	.89649	.803	-2.2275
	day8	2.00000*	.89649	.050	.0025
	day9	.61000	.89649	.512	-1.3875
	day10	.62000	.89649	.505	-1.3775

Multiple Comparisons

Dependent Variable: Inhibition

(I) Incubation	(J) Incubation	95% Confidence Upper Bound
day4	day1	25.3125
	day2	25.3125
	day3	8.9925
	day5	-2.9125
	day6	-2.9175
	day7	-2.6875
	day8	-.6875
	day9	-2.0775
	day10	-2.0675
day5	day1	30.2225
	day2	30.2225
	day3	13.9025
	day4	6.9075
	day6	1.9925
	day7	2.2225
	day8	4.2225
	day9	2.8325
	day10	2.8425
day6	day1	30.2275
	day2	30.2275
	day3	13.9075
	day4	6.9125
	day5	2.0025
	day7	2.2275
	day8	4.2275
	day9	2.8375
	day10	2.8475
day7	day1	29.9975
	day2	29.9975
	day3	13.6775
	day4	6.6825
	day5	1.7725
	day6	1.7675
	day8	3.9975
	day9	2.6075
	day10	2.6175

Multiple Comparisons

Dependent Variable: Inhibition

(I) Incubation	(J) Incubation	Mean Difference (I-J)	Std. Error	Sig.	95% ...
					Lower Bound
day8	day1	26.00000*	.89649	.000	24.0025
	day2	26.00000*	.89649	.000	24.0025
	day3	9.68000*	.89649	.000	7.6825
	day4	2.68500*	.89649	.013	.6875
	day5	-2.22500*	.89649	.032	-4.2225
	day6	-2.23000*	.89649	.032	-4.2275
	day7	-2.00000*	.89649	.050	-3.9975
	day9	-1.39000	.89649	.152	-3.3875
	day10	-1.38000	.89649	.155	-3.3775
day9	day1	27.39000*	.89649	.000	25.3925
	day2	27.39000*	.89649	.000	25.3925
	day3	11.07000*	.89649	.000	9.0725
	day4	4.07500*	.89649	.001	2.0775
	day5	-.83500	.89649	.374	-2.8325
	day6	-.84000	.89649	.371	-2.8375
	day7	-.61000	.89649	.512	-2.6075
	day8	1.39000	.89649	.152	-.6075
	day10	.01000	.89649	.991	-1.9875
day10	day1	27.38000*	.89649	.000	25.3825
	day2	27.38000*	.89649	.000	25.3825
	day3	11.06000*	.89649	.000	9.0625
	day4	4.06500*	.89649	.001	2.0675
	day5	-.84500	.89649	.368	-2.8425
	day6	-.85000	.89649	.365	-2.8475
	day7	-.62000	.89649	.505	-2.6175
	day8	1.38000	.89649	.155	-.6175
	day9	-.01000	.89649	.991	-2.0075

Multiple Comparisons

Dependent Variable: Inhibition

(I) Incubation	(J) Incubation	95% Confidence Upper Bound
day8	day1	27.9975
	day2	27.9975
	day3	11.6775
	day4	4.6825
	day5	-.2275
	day6	-.2325
	day7	-.0025
	day9	.6075
	day10	.6175
day9	day1	29.3875
	day2	29.3875
	day3	13.0675
	day4	6.0725
	day5	1.1625
	day6	1.1575
	day7	1.3875
	day8	3.3875
	day10	2.0075
day10	day1	29.3775
	day2	29.3775
	day3	13.0575
	day4	6.0625
	day5	1.1525
	day6	1.1475
	day7	1.3775
	day8	3.3775
	day9	1.9875

*. The mean difference is significant at the 0.05 level.

Homogeneous Subsets

Inhibition

	Incubation	N	Subset for alpha = 0.05				
			1	2	3	4	5
Duncan[a]	day1	2	.0000				
	day2	2	.0000				
	day3	2		16.3200			
	day4	2			23.3150		
	day8	2				26.0000	
	day10	2				27.3800	27.3800
	day9	2				27.3900	27.3900
	day7	2				28.0000	28.0000
	day5	2					28.2250
	day6	2					28.2300
	Sig.		1.000	1.000	1.000	.064	.401

Means for groups in homogeneous subsets are displayed.

a. Uses Harmonic Mean Sample Size = 2.000.
