# Supplementary material for: Biological characteristics of marine Streptomyces SK3 and optimization of cultivation conditions for production of compounds against Vibiriosis pathogen isolated from cultured white shrimp (Litopenaeus vannamei)
Source: PeerJ. 2024 Sep 24;12:e18053. doi: 10.7717/peerj.18053 (PMC11430173; doi:10.7717/peerj.18053)
Supplement: Supplemental Information 7 — Raw data exported from the statistical software SPSS (version 22) was analyzed using one-way ANOVA at a 95% confidence interval (p < 0.05). [file peerj-12-18053-s007.pdf]

```
 GET
  FILE='C:\Users\User\Desktop\paper SK3-ข้อมูล\ข้อมูลวิเคราะห์ spss\Agitation speed.
sav'.
DATASET NAME DataSet1 WINDOW=FRONT.
ONEWAY

Inhibition BY Agitation

/
STATISTICS
 DESCRIPTIVES EFFECTS
  /MISSING ANALYSIS
  /POSTHOC=DUNCAN LSD ALPHA(0.05).
```

Oneway

Notes

Output Created		07-MAY-2024 14:54:28
Comments		
Input	Data	C:\Users\User\Desktop\paper SK3-<br>\ s p s s \ A g i t a t i o n<br>speed.sav
	Active Dataset	DataSet1
	Filter	<none>
	Weight	<none>
	Split File	<none>
	N of Rows in Working Data File	10
Missing Value Handling	Definition of Missing	User-defined missing values are treated as missing.
	Cases Used	Statistics for each analysis are based on cases with no missing data for any variable in the analysis.
Syntax		ONEWAY Inhibition BY Agitation<br>/STATISTICS DESCRIPTIVES EFFECTS<br>/MISSING ANALYSIS<br>/POSTHOC=DUNCAN LSD ALPHA(0.05).
Resources	Processor Time	00:00:00.02
	Elapsed Time	00:00:00.05

```
[DataSet1] C:\Users\User\Desktop\paper SK3-ข้อมูล\ข้อมูลวิเคราะห์ spss\Agitation speed.sav
```

Descriptives

Inhibition

		N	Mean	Std. Deviation	Std. Error	95% Confidence ... Lower Bound
0		2	.0000	.00000	.00000	.0000
100		2	16.3150	.82731	.58500	8.8819
150		2	20.0000	.00000	.00000	20.0000
200		2	35.3200	.82024	.58000	27.9504
250		2	23.3400	.82024	.58000	15.9704
Total		10	18.9950	12.06834	3.81635	10.3618
Model	Fixed Effects			.63719	.20150	18.4770
	Random Effects				5.72008	3.1135

Descriptives

Inhibition

		95% Confidence Interval for Mean Upper Bound	Minimum	Maximum	Between-Component Variance
0		.0000	.00	.00	
100		23.7481	15.73	16.90	
150		20.0000	20.00	20.00	
200		42.6896	34.74	35.90	
250		30.7096	22.76	23.92	
Total		27.6282	.00	35.90	
Model	Fixed Effects	19.5130			
	Random Effects	34.8765			163.39377

ANOVA

Inhibition

	Sum of Squares	df	Mean Square	F	Sig.
Between Groups	1308.774	4	327.194	805.876	.000
Within Groups	2.030	5	.406		
Total	1310.804	9			

Post Hoc Tests

Multiple Comparisons

Dependent Variable: Inhibition

	(I) Agitation	(J) Agitation	Mean Difference (I-J)	Std. Error	Sig.	95% ...
						Lower Bound
LSD	0	100	-16.31500*	.63719	.000	-17.9529
		150	-20.00000*	.63719	.000	-21.6379
		200	-35.32000*	.63719	.000	-36.9579
		250	-23.34000*	.63719	.000	-24.9779
	100	0	16.31500*	.63719	.000	14.6771
		150	-3.68500*	.63719	.002	-5.3229
		200	-19.00500*	.63719	.000	-20.6429
		250	-7.02500*	.63719	.000	-8.6629
	150	0	20.00000*	.63719	.000	18.3621
		100	3.68500*	.63719	.002	2.0471
		200	-15.32000*	.63719	.000	-16.9579
		250	-3.34000*	.63719	.003	-4.9779
	200	0	35.32000*	.63719	.000	33.6821
		100	19.00500*	.63719	.000	17.3671
		150	15.32000*	.63719	.000	13.6821
		250	11.98000*	.63719	.000	10.3421
	250	0	23.34000*	.63719	.000	21.7021
		100	7.02500*	.63719	.000	5.3871
		150	3.34000*	.63719	.003	1.7021
		200	-11.98000*	.63719	.000	-13.6179

Multiple Comparisons

Dependent Variable: Inhibition

	(I) Agitation	(J) Agitation	95% Confidence Upper Bound
LSD	0	100	-14.6771
		150	-18.3621
		200	-33.6821
		250	-21.7021
	100	0	17.9529
		150	-2.0471
		200	-17.3671
		250	-5.3871
	150	0	21.6379
		100	5.3229
		200	-13.6821
		250	-1.7021
	200	0	36.9579
		100	20.6429
		150	16.9579
		250	13.6179
	250	0	24.9779
		100	8.6629
		150	4.9779
		200	-10.3421

*. The mean difference is significant at the 0.05 level.

Homogeneous Subsets

Inhibition

	Agitation	N	Subset for alpha = 0.05 1	2	3	4	5
Duncan[a]	0	2	.0000				
	100	2		16.3150			
	150	2			20.0000		
	250	2				23.3400	
	200	2					35.3200
	Sig.		1.000	1.000	1.000	1.000	1.000

Means for groups in homogeneous subsets are displayed.

a. Uses Harmonic Mean Sample Size = 2.000.
