# Supplementary material for: Biological characteristics of marine Streptomyces SK3 and optimization of cultivation conditions for production of compounds against Vibiriosis pathogen isolated from cultured white shrimp (Litopenaeus vannamei)
Source: PeerJ. 2024 Sep 24;12:e18053. doi: 10.7717/peerj.18053 (PMC11430173; doi:10.7717/peerj.18053)
Supplement: Supplemental Information 8 — Raw data exported from the statistical software SPSS (version 22) was analyzed using one-way ANOVA at a 95% confidence interval (p < 0.05) of nitrogen sources. [file peerj-12-18053-s008.pdf]

```
ONEWAY Inhibition BY Nitrogen
  /STATISTICS DESCRIPTIVES EFFECTS
  /MISSING ANALYSIS
  /POSTHOC=DUNCAN LSD ALPHA(0.05).
```

Oneway

Notes

Output Created		27-APR-2024 14:15:50
Comments		
Input	Data	C:\Users\User\Desktop\paper SK3-\ spss\Nitrogen sources.sav
	Active Dataset	DataSet1
	Filter	<none>
	Weight	<none>
	Split File	<none>
	N of Rows in Working Data File	12
Missing Value Handling	Definition of Missing	User-defined missing values are treated as missing.
	Cases Used	Statistics for each analysis are based on cases with no missing data for any variable in the analysis.
Syntax		ONEWAY Inhibition BY Nitrogen /STATISTICS DESCRIPTIVES EFFECTS /MISSING ANALYSIS /POSTHOC=DUNCAN LSD ALPHA(0.05).
Resources	Processor Time	00:00:00.08
	Elapsed Time	00:00:00.06

[DataSet1] C:\Users\User\Desktop\paper SK3-ข้อมูล\ข้อมูลวิเคราะห์ spss\Nitrogen sources.sav

Descriptives

Inhibition

		N	Mean	Std. Deviation	Std. Error	95% Confidence ...
						Lower Bound
Casein		2	22.6700	.00000	.00000	22.6700
Peptone		2	21.6650	.71418	.50500	15.2484
Beef extract		2	20.6700	.00000	.00000	20.6700
Malt extract		2	23.3300	1.41421	1.00000	10.6238
Urea		2	18.0000	.82024	.58000	10.6304
No supplement		2	32.6700	.82024	.58000	25.3004
Total		12	23.1675	4.81847	1.39097	20.1060
Model	Fixed Effects			.80163	.23141	22.6013
	Random Effects				2.04751	17.9042

Descriptives

Inhibition

		95% Confidence Interval for Mean	Minimum	Maximum	Between-Component Variance
		Upper Bound			
Casein		22.6700	22.67	22.67	
Peptone		28.0816	21.16	22.17	
Beef extract		20.6700	20.67	20.67	
Malt extract		36.0362	22.33	24.33	
Urea		25.3696	17.42	18.58	
No supplement		40.0396	32.09	33.25	
Total		26.2290	17.42	33.25	
Model	Fixed Effects	23.7337			
	Random Effects	28.4308			24.83259

ANOVA

Inhibition

	Sum of Squares	df	Mean Square	F	Sig.
Between Groups	251.539	5	50.308	78.287	.000
Within Groups	3.856	6	.643		
Total	255.395	11			

Post Hoc Tests

Multiple Comparisons

Dependent Variable: Inhibition

	(I) Nitrogen	(J) Nitrogen	Mean Difference (I-J)	Std. Error	Sig.	95% ...
						Lower Bound
LSD	Casein	Peptone	1.00500	.80163	.257	-.9565
		Beef extract	2.00000*	.80163	.047	.0385
		Malt extract	-.66000	.80163	.442	-2.6215
		Urea	4.67000*	.80163	.001	2.7085
		No supplement	-10.00000*	.80163	.000	-11.9615
	Peptone	Casein	-1.00500	.80163	.257	-2.9665
		Beef extract	.99500	.80163	.261	-.9665
		Malt extract	-1.66500	.80163	.083	-3.6265
		Urea	3.66500*	.80163	.004	1.7035
		No supplement	-11.00500*	.80163	.000	-12.9665
	Beef extract	Casein	-2.00000*	.80163	.047	-3.9615
		Peptone	-.99500	.80163	.261	-2.9565
		Malt extract	-2.66000*	.80163	.016	-4.6215
		Urea	2.67000*	.80163	.016	.7085
		No supplement	-12.00000*	.80163	.000	-13.9615
	Malt extract	Casein	.66000	.80163	.442	-1.3015
		Peptone	1.66500	.80163	.083	-.2965
		Beef extract	2.66000*	.80163	.016	.6985
		Urea	5.33000*	.80163	.001	3.3685
		No supplement	-9.34000*	.80163	.000	-11.3015
	Urea	Casein	-4.67000*	.80163	.001	-6.6315
		Peptone	-3.66500*	.80163	.004	-5.6265
		Beef extract	-2.67000*	.80163	.016	-4.6315
		Malt extract	-5.33000*	.80163	.001	-7.2915
		No supplement	-14.67000*	.80163	.000	-16.6315
	No supplement	Casein	10.00000*	.80163	.000	8.0385
		Peptone	11.00500*	.80163	.000	9.0435
		Beef extract	12.00000*	.80163	.000	10.0385
		Malt extract	9.34000*	.80163	.000	7.3785
		Urea	14.67000*	.80163	.000	12.7085

Multiple Comparisons

Dependent Variable: Inhibition

			95% Confidence
	(I) Nitrogen	(J) Nitrogen	Upper Bound
LSD	Casein	Peptone	2.9665
		Beef extract	3.9615
		Malt extract	1.3015
		Urea	6.6315
		No supplement	-8.0385
	Peptone	Casein	.9565
		Beef extract	2.9565
		Malt extract	.2965
		Urea	5.6265
		No supplement	-9.0435
	Beef extract	Casein	-.0385
		Peptone	.9665
		Malt extract	-.6985
		Urea	4.6315
		No supplement	-10.0385
	Malt extract	Casein	2.6215
		Peptone	3.6265
		Beef extract	4.6215
		Urea	7.2915
		No supplement	-7.3785
	Urea	Casein	-2.7085
		Peptone	-1.7035
		Beef extract	-.7085
		Malt extract	-3.3685
		No supplement	-12.7085
	No supplement	Casein	11.9615
		Peptone	12.9665
		Beef extract	13.9615
		Malt extract	11.3015
		Urea	16.6315

*. The mean difference is significant at the 0.05 level.

Homogeneous Subsets

Inhibition

	Nitrogen	N	Subset for alpha = 0.05			
			1	2	3	4
Duncan[a]	Urea	2	18.0000			
	Beef extract	2		20.6700		
	Peptone	2		21.6650	21.6650	
	Casein	2		22.6700	22.6700	
	Malt extract	2			23.3300	
	No supplement	2				32.6700
	Sig.		1.000	.053	.092	1.000

Means for groups in homogeneous subsets are displayed.

a. Uses Harmonic Mean Sample Size = 2.000.
