# Supplementary material for: Biological characteristics of marine Streptomyces SK3 and optimization of cultivation conditions for production of compounds against Vibiriosis pathogen isolated from cultured white shrimp (Litopenaeus vannamei)
Source: PeerJ. 2024 Sep 24;12:e18053. doi: 10.7717/peerj.18053 (PMC11430173; doi:10.7717/peerj.18053)
Supplement: Supplemental Information 9 — Raw data exported from the statistical software SPSS (version 22) was analyzed using one-way ANOVA at a 95% confidence interval (p < 0.05) of pH. [file peerj-12-18053-s009.pdf]

```
 GET
  FILE='C:\Users\User\Desktop\paper SK3-ข้อมูล\ข้อมูลวิเคราะห์ spss\pH.sav'.
DATASET NAME DataSet1 WINDOW=FRONT.
ONEWAY Inhibition BY pH

/
STATISTICS
 DESCRIPTIVES EFFECTS
  /MISSING ANALYSIS
  /POSTHOC=DUNCAN LSD ALPHA(0.05).
```

Oneway

Notes

Output Created		27-APR-2024 13:22:33
Comments		
Input	Data	C:\Users\User\Desktop\paper SK3-\ spss\pH.sav
	Active Dataset	DataSet1
	Filter	<none>
	Weight	<none>
	Split File	<none>
	N of Rows in Working Data File	20
Missing Value Handling	Definition of Missing	User-defined missing values are treated as missing.
	Cases Used	Statistics for each analysis are based on cases with no missing data for any variable in the analysis.
Syntax		ONEWAY Inhibition BY pH /STATISTICS DESCRIPTIVES EFFECTS /MISSING ANALYSIS /POSTHOC=DUNCAN LSD ALPHA(0.05).
Resources	Processor Time	00:00:00.02
	Elapsed Time	00:00:00.09

```
[DataSet1] C:\Users\User\Desktop\paper SK3-ข้อมูล\ข้อมูลวิเคราะห์ spss\pH.sav
```

Descriptives

Inhibition

		N	Mean	Std. Deviation	Std. Error	95% Confidence ... Lower Bound
3		2	.0000	.00000	.00000	.0000
4		2	.0000	.00000	.00000	.0000
5		2	14.5650	.81317	.57500	7.2589
6		2	28.3650	.82731	.58500	20.9319
7		2	33.0000	.00000	.00000	33.0000
8		2	27.3300	.82024	.58000	19.9604
9		2	23.0000	.00000	.00000	23.0000
10		2	18.3300	.82024	.58000	10.9604
11		2	15.6700	.82024	.58000	8.3004
12		2	.0000	.00000	.00000	.0000
Total		20	16.0260	12.12451	2.71112	10.3516
Model	Fixed Effects			.58001	.12969	15.7370
	Random Effects				3.93680	7.1203

Descriptives

Inhibition

		95% Confidence Interval for Mean Upper Bound	Minimum	Maximum	Between-Component Variance
3		.0000	.00	.00	
4		.0000	.00	.00	
5		21.8711	13.99	15.14	
6		35.7981	27.78	28.95	
7		33.0000	33.00	33.00	
8		34.6996	26.75	27.91	
9		23.0000	23.00	23.00	
10		25.6996	17.75	18.91	
11		23.0396	15.09	16.25	
12		.0000	.00	.00	
Total		21.7004	.00	33.00	
Model	Fixed Effects	16.3150			
	Random Effects	24.9317			154.81539

ANOVA

Inhibition

	Sum of Squares	df	Mean Square	F	Sig.
Between Groups	2789.705	9	309.967	921.397	.000
Within Groups	3.364	10	.336		
Total	2793.069	19			

Post Hoc Tests

Multiple Comparisons

Dependent Variable: Inhibition

	(I) pH	(J) pH	Mean Difference (I-J)	Std. Error	Sig.	95% Confidence Interval	
						Lower Bound	Upper Bound
LSD	3	4	.00000	.58001	1.000	-1.2923	1.2923
		5	-14.56500*	.58001	.000	-15.8573	-13.2727
		6	-28.36500*	.58001	.000	-29.6573	-27.0727
		7	-33.00000*	.58001	.000	-34.2923	-31.7077
		8	-27.33000*	.58001	.000	-28.6223	-26.0377
		9	-23.00000*	.58001	.000	-24.2923	-21.7077
		10	-18.33000*	.58001	.000	-19.6223	-17.0377
		11	-15.67000*	.58001	.000	-16.9623	-14.3777
		12	.00000	.58001	1.000	-1.2923	1.2923
	4	3	.00000	.58001	1.000	-1.2923	1.2923
		5	-14.56500*	.58001	.000	-15.8573	-13.2727
		6	-28.36500*	.58001	.000	-29.6573	-27.0727
		7	-33.00000*	.58001	.000	-34.2923	-31.7077
		8	-27.33000*	.58001	.000	-28.6223	-26.0377
		9	-23.00000*	.58001	.000	-24.2923	-21.7077
		10	-18.33000*	.58001	.000	-19.6223	-17.0377
		11	-15.67000*	.58001	.000	-16.9623	-14.3777
		12	.00000	.58001	1.000	-1.2923	1.2923
	5	3	14.56500*	.58001	.000	13.2727	15.8573
		4	14.56500*	.58001	.000	13.2727	15.8573
		6	-13.80000*	.58001	.000	-15.0923	-12.5077
		7	-18.43500*	.58001	.000	-19.7273	-17.1427
		8	-12.76500*	.58001	.000	-14.0573	-11.4727
		9	-8.43500*	.58001	.000	-9.7273	-7.1427
		10	-3.76500*	.58001	.000	-5.0573	-2.4727
		11	-1.10500	.58001	.086	-2.3973	.1873
		12	14.56500*	.58001	.000	13.2727	15.8573

Multiple Comparisons

Dependent Variable: Inhibition

(I) pH	(J) pH	Mean Difference (I-J)	Std. Error	Sig.	95% Confidence Interval Lower Bound	Upper Bound
6	3	28.36500*	.58001	.000	27.0727	29.6573
	4	28.36500*	.58001	.000	27.0727	29.6573
	5	13.80000*	.58001	.000	12.5077	15.0923
	7	-4.63500*	.58001	.000	-5.9273	-3.3427
	8	1.03500	.58001	.105	-.2573	2.3273
	9	5.36500*	.58001	.000	4.0727	6.6573
	10	10.03500*	.58001	.000	8.7427	11.3273
	11	12.69500*	.58001	.000	11.4027	13.9873
	12	28.36500*	.58001	.000	27.0727	29.6573
7	3	33.00000*	.58001	.000	31.7077	34.2923
	4	33.00000*	.58001	.000	31.7077	34.2923
	5	18.43500*	.58001	.000	17.1427	19.7273
	6	4.63500*	.58001	.000	3.3427	5.9273
	8	5.67000*	.58001	.000	4.3777	6.9623
	9	10.00000*	.58001	.000	8.7077	11.2923
	10	14.67000*	.58001	.000	13.3777	15.9623
	11	17.33000*	.58001	.000	16.0377	18.6223
	12	33.00000*	.58001	.000	31.7077	34.2923
8	3	27.33000*	.58001	.000	26.0377	28.6223
	4	27.33000*	.58001	.000	26.0377	28.6223
	5	12.76500*	.58001	.000	11.4727	14.0573
	6	-1.03500	.58001	.105	-2.3273	.2573
	7	-5.67000*	.58001	.000	-6.9623	-4.3777
	9	4.33000*	.58001	.000	3.0377	5.6223
	10	9.00000*	.58001	.000	7.7077	10.2923
	11	11.66000*	.58001	.000	10.3677	12.9523
	12	27.33000*	.58001	.000	26.0377	28.6223
9	3	23.00000*	.58001	.000	21.7077	24.2923
	4	23.00000*	.58001	.000	21.7077	24.2923
	5	8.43500*	.58001	.000	7.1427	9.7273
	6	-5.36500*	.58001	.000	-6.6573	-4.0727
	7	-10.00000*	.58001	.000	-11.2923	-8.7077
	8	-4.33000*	.58001	.000	-5.6223	-3.0377
	10	4.67000*	.58001	.000	3.3777	5.9623
	11	7.33000*	.58001	.000	6.0377	8.6223
	12	23.00000*	.58001	.000	21.7077	24.2923

Multiple Comparisons

Dependent Variable: Inhibition

(I) pH	(J) pH	Mean Difference (I-J)	Std. Error	Sig.	95% Confidence Interval	
					Lower Bound	Upper Bound
10	3	18.33000*	.58001	.000	17.0377	19.6223
	4	18.33000*	.58001	.000	17.0377	19.6223
	5	3.76500*	.58001	.000	2.4727	5.0573
	6	-10.03500*	.58001	.000	-11.3273	-8.7427
	7	-14.67000*	.58001	.000	-15.9623	-13.3777
	8	-9.00000*	.58001	.000	-10.2923	-7.7077
	9	-4.67000*	.58001	.000	-5.9623	-3.3777
	11	2.66000*	.58001	.001	1.3677	3.9523
	12	18.33000*	.58001	.000	17.0377	19.6223
11	3	15.67000*	.58001	.000	14.3777	16.9623
	4	15.67000*	.58001	.000	14.3777	16.9623
	5	1.10500	.58001	.086	-.1873	2.3973
	6	-12.69500*	.58001	.000	-13.9873	-11.4027
	7	-17.33000*	.58001	.000	-18.6223	-16.0377
	8	-11.66000*	.58001	.000	-12.9523	-10.3677
	9	-7.33000*	.58001	.000	-8.6223	-6.0377
	10	-2.66000*	.58001	.001	-3.9523	-1.3677
	12	15.67000*	.58001	.000	14.3777	16.9623
12	3	.00000	.58001	1.000	-1.2923	1.2923
	4	.00000	.58001	1.000	-1.2923	1.2923
	5	-14.56500*	.58001	.000	-15.8573	-13.2727
	6	-28.36500*	.58001	.000	-29.6573	-27.0727
	7	-33.00000*	.58001	.000	-34.2923	-31.7077
	8	-27.33000*	.58001	.000	-28.6223	-26.0377
	9	-23.00000*	.58001	.000	-24.2923	-21.7077
	10	-18.33000*	.58001	.000	-19.6223	-17.0377
	11	-15.67000*	.58001	.000	-16.9623	-14.3777

*. The mean difference is significant at the 0.05 level.

Homogeneous Subsets

Inhibition

	pH	N	Subset for alpha = 0.05					
			1	2	3	4	5	6
Duncan[a]	3	2	.0000					
	4	2	.0000					
	12	2	.0000					
	5	2		14.5650				
	11	2		15.6700				
	10	2			18.3300			
	9	2				23.0000		
	8	2					27.3300	
	6	2					28.3650	
	7	2						33.0000
	Sig.		1.000	.086	1.000	1.000	.105	1.000

Means for groups in homogeneous subsets are displayed.

a. Uses Harmonic Mean Sample Size = 2.000.
