# Supplementary material for: Biological characteristics of marine Streptomyces SK3 and optimization of cultivation conditions for production of compounds against Vibiriosis pathogen isolated from cultured white shrimp (Litopenaeus vannamei)
Source: PeerJ. 2024 Sep 24;12:e18053. doi: 10.7717/peerj.18053 (PMC11430173; doi:10.7717/peerj.18053)
Supplement: Supplemental Information 10 — Raw data exported from the statistical software SPSS (version 22) was analyzed using one-way ANOVA at a 95% confidence interval (p < 0.05) of medium formula. [file peerj-12-18053-s010.pdf]

```
ONEWAY Inhibition BY Medium2
  /STATISTICS DESCRIPTIVES EFFECTS
  /MISSING ANALYSIS
  /POSTHOC=DUNCAN LSD ALPHA(0.05).
```

Oneway

Notes

Output Created		27-APR-2024 10:52:31
Comments		
Input	Active Dataset	DataSet0
	Filter	<none>
	Weight	<none>
	Split File	<none>
	N of Rows in Working Data File	24
Missing Value Handling	Definition of Missing	User-defined missing values are treated as missing.
	Cases Used	Statistics for each analysis are based on cases with no missing data for any variable in the analysis.
Syntax		ONEWAY Inhibition BY Medium2<br>/STATISTICS DESCRIPTIVES EFFECTS<br>/MISSING ANALYSIS<br>/POSTHOC=DUNCAN LSD ALPHA(0.05).
Resources	Processor Time	00:00:00.08
	Elapsed Time	00:00:00.19

```
[DataSet0]
```

Descriptives

Inhibition

		N	Mean	Std. Deviation	Std. Error	95% Confidence ...
						Lower Bound
YM		2	28.0000	.00000	.00000	28.0000
YM/2		2	34.0000	.00000	.00000	34.0000
YM/3		2	24.0800	.82024	.58000	16.7104
YM/4		2	18.0800	.82024	.58000	10.7104
YM/5		2	11.6450	1.61927	1.14500	-2.9036
YM/6		2	11.3300	.00000	.00000	11.3300
ISP3		2	20.6450	1.61927	1.14500	6.0964
ISP3/2		2	26.0800	.82024	.58000	18.7104
ISP3/3		2	13.6450	1.61927	1.14500	-.9036
ISP3/4		2	11.6450	1.61927	1.14500	-2.9036
ISP3/5		2	.0000	.00000	.00000	.0000
ISP3/6		2	.0000	.00000	.00000	.0000
Total		24	16.5958	10.38425	2.11968	12.2109
Model	Fixed Effects			1.02089	.20839	16.1418
	Random Effects				3.05731	9.8667

Descriptives

Inhibition

		95% Confidence Interval for Mean	Minimum	Maximum	Between-Component Variance
		Upper Bound			
YM		28.0000	28.00	28.00	
YM/2		34.0000	34.00	34.00	
YM/3		31.4496	23.50	24.66	
YM/4		25.4496	17.50	18.66	
YM/5		26.1936	10.50	12.79	
YM/6		11.3300	11.33	11.33	
ISP3		35.1936	19.50	21.79	
ISP3/2		33.4496	25.50	26.66	
ISP3/3		28.1936	12.50	14.79	
ISP3/4		26.1936	10.50	12.79	
ISP3/5		.0000	.00	.00	
ISP3/6		.0000	.00	.00	
Total		20.9807	.00	34.00	
Model	Fixed Effects	17.0499			
	Random Effects	23.3249			111.64453

ANOVA

Inhibition

	Sum of Squares	df	Mean Square	F	Sig.
Between Groups	2467.644	11	224.331	215.244	.000
Within Groups	12.507	12	1.042		
Total	2480.151	23			

Post Hoc Tests

Multiple Comparisons

Dependent Variable: Inhibition

						95% ...
	(I) Medium2	(J) Medium2	Mean Difference (I-J)	Std. Error	Sig.	Lower Bound
LSD	YM	YM/2	-6.00000*	1.02089	.000	-8.2243
		YM/3	3.92000*	1.02089	.002	1.6957
		YM/4	9.92000*	1.02089	.000	7.6957
		YM/5	16.35500*	1.02089	.000	14.1307
		YM/6	16.67000*	1.02089	.000	14.4457
		ISP3	7.35500*	1.02089	.000	5.1307
		ISP3/2	1.92000	1.02089	.084	-.3043
		ISP3/3	14.35500*	1.02089	.000	12.1307
		ISP3/4	16.35500*	1.02089	.000	14.1307
		ISP3/5	28.00000*	1.02089	.000	25.7757
		ISP3/6	28.00000*	1.02089	.000	25.7757
	YM/2	YM	6.00000*	1.02089	.000	3.7757
		YM/3	9.92000*	1.02089	.000	7.6957
		YM/4	15.92000*	1.02089	.000	13.6957
		YM/5	22.35500*	1.02089	.000	20.1307
		YM/6	22.67000*	1.02089	.000	20.4457
		ISP3	13.35500*	1.02089	.000	11.1307
		ISP3/2	7.92000*	1.02089	.000	5.6957
		ISP3/3	20.35500*	1.02089	.000	18.1307
		ISP3/4	22.35500*	1.02089	.000	20.1307
		ISP3/5	34.00000*	1.02089	.000	31.7757
		ISP3/6	34.00000*	1.02089	.000	31.7757
	YM/3	YM	-3.92000*	1.02089	.002	-6.1443
		YM/2	-9.92000*	1.02089	.000	-12.1443
		YM/4	6.00000*	1.02089	.000	3.7757
		YM/5	12.43500*	1.02089	.000	10.2107
		YM/6	12.75000*	1.02089	.000	10.5257

Multiple Comparisons

Dependent Variable: Inhibition

	(I) Medium2	(J) Medium2	95% Confidence Upper Bound
LSD	YM	YM/2	-3.7757
		YM/3	6.1443
		YM/4	12.1443
		YM/5	18.5793
		YM/6	18.8943
		ISP3	9.5793
		ISP3/2	4.1443
		ISP3/3	16.5793
		ISP3/4	18.5793
		ISP3/5	30.2243
		ISP3/6	30.2243
	YM/2	YM	8.2243
		YM/3	12.1443
		YM/4	18.1443
		YM/5	24.5793
		YM/6	24.8943
		ISP3	15.5793
		ISP3/2	10.1443
		ISP3/3	22.5793
		ISP3/4	24.5793
		ISP3/5	36.2243
		ISP3/6	36.2243
	YM/3	YM	-1.6957
		YM/2	-7.6957
		YM/4	8.2243
		YM/5	14.6593
		YM/6	14.9743

Multiple Comparisons

Dependent Variable: Inhibition

(I) Medium2	(J) Medium2	Mean Difference (I-J)	Std. Error	Sig.	95% ... Lower Bound
	ISP3	3.43500*	1.02089	.006	1.2107
	ISP3/2	-2.00000	1.02089	.074	-4.2243
	ISP3/3	10.43500*	1.02089	.000	8.2107
	ISP3/4	12.43500*	1.02089	.000	10.2107
	ISP3/5	24.08000*	1.02089	.000	21.8557
	ISP3/6	24.08000*	1.02089	.000	21.8557
YM/4	YM	-9.92000*	1.02089	.000	-12.1443
	YM/2	-15.92000*	1.02089	.000	-18.1443
	YM/3	-6.00000*	1.02089	.000	-8.2243
	YM/5	6.43500*	1.02089	.000	4.2107
	YM/6	6.75000*	1.02089	.000	4.5257
	ISP3	-2.56500*	1.02089	.027	-4.7893
	ISP3/2	-8.00000*	1.02089	.000	-10.2243
	ISP3/3	4.43500*	1.02089	.001	2.2107
	ISP3/4	6.43500*	1.02089	.000	4.2107
	ISP3/5	18.08000*	1.02089	.000	15.8557
	ISP3/6	18.08000*	1.02089	.000	15.8557
YM/5	YM	-16.35500*	1.02089	.000	-18.5793
	YM/2	-22.35500*	1.02089	.000	-24.5793
	YM/3	-12.43500*	1.02089	.000	-14.6593
	YM/4	-6.43500*	1.02089	.000	-8.6593
	YM/6	.31500	1.02089	.763	-1.9093
	ISP3	-9.00000*	1.02089	.000	-11.2243
	ISP3/2	-14.43500*	1.02089	.000	-16.6593
	ISP3/3	-2.00000	1.02089	.074	-4.2243
	ISP3/4	.00000	1.02089	1.000	-2.2243
	ISP3/5	11.64500*	1.02089	.000	9.4207
	ISP3/6	11.64500*	1.02089	.000	9.4207
YM/6	YM	-16.67000*	1.02089	.000	-18.8943
	YM/2	-22.67000*	1.02089	.000	-24.8943
	YM/3	-12.75000*	1.02089	.000	-14.9743
	YM/4	-6.75000*	1.02089	.000	-8.9743
	YM/5	-.31500	1.02089	.763	-2.5393
	ISP3	-9.31500*	1.02089	.000	-11.5393
	ISP3/2	-14.75000*	1.02089	.000	-16.9743
	ISP3/3	-2.31500*	1.02089	.043	-4.5393
	ISP3/4	-.31500	1.02089	.763	-2.5393

Multiple Comparisons

Dependent Variable: Inhibition

(I) Medium2	(J) Medium2	95% Confidence Upper Bound
	ISP3	5.6593
	ISP3/2	.2243
	ISP3/3	12.6593
	ISP3/4	14.6593
	ISP3/5	26.3043
	ISP3/6	26.3043
YM/4	YM	-7.6957
	YM/2	-13.6957
	YM/3	-3.7757
	YM/5	8.6593
	YM/6	8.9743
	ISP3	-.3407
	ISP3/2	-5.7757
	ISP3/3	6.6593
	ISP3/4	8.6593
	ISP3/5	20.3043
	ISP3/6	20.3043
YM/5	YM	-14.1307
	YM/2	-20.1307
	YM/3	-10.2107
	YM/4	-4.2107
	YM/6	2.5393
	ISP3	-6.7757
	ISP3/2	-12.2107
	ISP3/3	.2243
	ISP3/4	2.2243
	ISP3/5	13.8693
	ISP3/6	13.8693
YM/6	YM	-14.4457
	YM/2	-20.4457
	YM/3	-10.5257
	YM/4	-4.5257
	YM/5	1.9093
	ISP3	-7.0907
	ISP3/2	-12.5257
	ISP3/3	-.0907
	ISP3/4	1.9093

Multiple Comparisons

Dependent Variable: Inhibition

(I) Medium2	(J) Medium2	Mean Difference (I-J)	Std. Error	Sig.	95% ... Lower Bound
	ISP3/5	11.33000*	1.02089	.000	9.1057
	ISP3/6	11.33000*	1.02089	.000	9.1057
ISP3	YM	-7.35500*	1.02089	.000	-9.5793
	YM/2	-13.35500*	1.02089	.000	-15.5793
	YM/3	-3.43500*	1.02089	.006	-5.6593
	YM/4	2.56500*	1.02089	.027	.3407
	YM/5	9.00000*	1.02089	.000	6.7757
	YM/6	9.31500*	1.02089	.000	7.0907
	ISP3/2	-5.43500*	1.02089	.000	-7.6593
	ISP3/3	7.00000*	1.02089	.000	4.7757
	ISP3/4	9.00000*	1.02089	.000	6.7757
	ISP3/5	20.64500*	1.02089	.000	18.4207
	ISP3/6	20.64500*	1.02089	.000	18.4207
ISP3/2	YM	-1.92000	1.02089	.084	-4.1443
	YM/2	-7.92000*	1.02089	.000	-10.1443
	YM/3	2.00000	1.02089	.074	-.2243
	YM/4	8.00000*	1.02089	.000	5.7757
	YM/5	14.43500*	1.02089	.000	12.2107
	YM/6	14.75000*	1.02089	.000	12.5257
	ISP3	5.43500*	1.02089	.000	3.2107
	ISP3/3	12.43500*	1.02089	.000	10.2107
	ISP3/4	14.43500*	1.02089	.000	12.2107
	ISP3/5	26.08000*	1.02089	.000	23.8557
	ISP3/6	26.08000*	1.02089	.000	23.8557
ISP3/3	YM	-14.35500*	1.02089	.000	-16.5793
	YM/2	-20.35500*	1.02089	.000	-22.5793
	YM/3	-10.43500*	1.02089	.000	-12.6593
	YM/4	-4.43500*	1.02089	.001	-6.6593
	YM/5	2.00000	1.02089	.074	-.2243
	YM/6	2.31500*	1.02089	.043	.0907
	ISP3	-7.00000*	1.02089	.000	-9.2243
	ISP3/2	-12.43500*	1.02089	.000	-14.6593
	ISP3/4	2.00000	1.02089	.074	-.2243
	ISP3/5	13.64500*	1.02089	.000	11.4207
	ISP3/6	13.64500*	1.02089	.000	11.4207
ISP3/4	YM	-16.35500*	1.02089	.000	-18.5793
	YM/2	-22.35500*	1.02089	.000	-24.5793

Multiple Comparisons

Dependent Variable: Inhibition

(I) Medium2	(J) Medium2	95% Confidence Upper Bound
	ISP3/5	13.5543
	ISP3/6	13.5543
ISP3	YM	-5.1307
	YM/2	-11.1307
	YM/3	-1.2107
	YM/4	4.7893
	YM/5	11.2243
	YM/6	11.5393
	ISP3/2	-3.2107
	ISP3/3	9.2243
	ISP3/4	11.2243
	ISP3/5	22.8693
	ISP3/6	22.8693
ISP3/2	YM	.3043
	YM/2	-5.6957
	YM/3	4.2243
	YM/4	10.2243
	YM/5	16.6593
	YM/6	16.9743
	ISP3	7.6593
	ISP3/3	14.6593
	ISP3/4	16.6593
	ISP3/5	28.3043
	ISP3/6	28.3043
ISP3/3	YM	-12.1307
	YM/2	-18.1307
	YM/3	-8.2107
	YM/4	-2.2107
	YM/5	4.2243
	YM/6	4.5393
	ISP3	-4.7757
	ISP3/2	-10.2107
	ISP3/4	4.2243
	ISP3/5	15.8693
	ISP3/6	15.8693
ISP3/4	YM	-14.1307
	YM/2	-20.1307

Multiple Comparisons

Dependent Variable: Inhibition

(I) Medium2	(J) Medium2	Mean Difference (I-J)	Std. Error	Sig.	95% ... Lower Bound
	YM/3	-12.43500*	1.02089	.000	-14.6593
	YM/4	-6.43500*	1.02089	.000	-8.6593
	YM/5	.00000	1.02089	1.000	-2.2243
	YM/6	.31500	1.02089	.763	-1.9093
	ISP3	-9.00000*	1.02089	.000	-11.2243
	ISP3/2	-14.43500*	1.02089	.000	-16.6593
	ISP3/3	-2.00000	1.02089	.074	-4.2243
	ISP3/5	11.64500*	1.02089	.000	9.4207
	ISP3/6	11.64500*	1.02089	.000	9.4207
ISP3/5	YM	-28.00000*	1.02089	.000	-30.2243
	YM/2	-34.00000*	1.02089	.000	-36.2243
	YM/3	-24.08000*	1.02089	.000	-26.3043
	YM/4	-18.08000*	1.02089	.000	-20.3043
	YM/5	-11.64500*	1.02089	.000	-13.8693
	YM/6	-11.33000*	1.02089	.000	-13.5543
	ISP3	-20.64500*	1.02089	.000	-22.8693
	ISP3/2	-26.08000*	1.02089	.000	-28.3043
	ISP3/3	-13.64500*	1.02089	.000	-15.8693
	ISP3/4	-11.64500*	1.02089	.000	-13.8693
	ISP3/6	.00000	1.02089	1.000	-2.2243
ISP3/6	YM	-28.00000*	1.02089	.000	-30.2243
	YM/2	-34.00000*	1.02089	.000	-36.2243
	YM/3	-24.08000*	1.02089	.000	-26.3043
	YM/4	-18.08000*	1.02089	.000	-20.3043
	YM/5	-11.64500*	1.02089	.000	-13.8693
	YM/6	-11.33000*	1.02089	.000	-13.5543
	ISP3	-20.64500*	1.02089	.000	-22.8693
	ISP3/2	-26.08000*	1.02089	.000	-28.3043
	ISP3/3	-13.64500*	1.02089	.000	-15.8693
	ISP3/4	-11.64500*	1.02089	.000	-13.8693
	ISP3/5	.00000	1.02089	1.000	-2.2243

Multiple Comparisons

Dependent Variable: Inhibition

(I) Medium2	(J) Medium2	95% Confidence Upper Bound
	YM/3	-10.2107
	YM/4	-4.2107
	YM/5	2.2243
	YM/6	2.5393
	ISP3	-6.7757
	ISP3/2	-12.2107
	ISP3/3	.2243
	ISP3/5	13.8693
	ISP3/6	13.8693
ISP3/5	YM	-25.7757
	YM/2	-31.7757
	YM/3	-21.8557
	YM/4	-15.8557
	YM/5	-9.4207
	YM/6	-9.1057
	ISP3	-18.4207
	ISP3/2	-23.8557
	ISP3/3	-11.4207
	ISP3/4	-9.4207
	ISP3/6	2.2243
ISP3/6	YM	-25.7757
	YM/2	-31.7757
	YM/3	-21.8557
	YM/4	-15.8557
	YM/5	-9.4207
	YM/6	-9.1057
	ISP3	-18.4207
	ISP3/2	-23.8557
	ISP3/3	-11.4207
	ISP3/4	-9.4207
	ISP3/5	2.2243

*. The mean difference is significant at the 0.05 level.

Homogeneous Subsets

Inhibition

	Medium2	N	Subset for alpha = 0.05						
			1	2	3	4	5	6	7
Duncan[a]	ISP3/5	2	.0000						
	ISP3/6	2	.0000						
	YM/6	2		11.3300					
	YM/5	2		11.6450					
	ISP3/4	2		11.6450					
	ISP3/3	2		13.6450					
	YM/4	2			18.0800				
	ISP3	2				20.6450			
	YM/3	2					24.0800		
	ISP3/2	2					26.0800	26.0800	
	YM	2						28.0000	
	YM/2	2							34.0000
	Sig.		1.000	.057	1.000	1.000	.074	.084	1.000

Means for groups in homogeneous subsets are displayed.

a. Uses Harmonic Mean Sample Size = 2.000.
