# Supplementary material for: Biological characteristics of marine Streptomyces SK3 and optimization of cultivation conditions for production of compounds against Vibiriosis pathogen isolated from cultured white shrimp (Litopenaeus vannamei)
Source: PeerJ. 2024 Sep 24;12:e18053. doi: 10.7717/peerj.18053 (PMC11430173; doi:10.7717/peerj.18053)
Supplement: Supplemental Information 11 — Raw data exported from the statistical software SPSS (version 22) was analyzed using one-way ANOVA at a 95% confidence interval (p < 0.05) of salt concentration. [file peerj-12-18053-s011.pdf]

```
ONEWAY Inhibition BY Salt
  /STATISTICS DESCRIPTIVES EFFECTS
  /MISSING ANALYSIS
  /POSTHOC=DUNCAN LSD ALPHA(0.05).
```

Oneway

Notes

Output Created		03-MAY-2024 17:12:22
Comments		
Input	Active Dataset	DataSet0
	Filter	<none>
	Weight	<none>
	Split File	<none>
	N of Rows in Working Data File	14
Missing Value Handling	Definition of Missing	User-defined missing values are treated as missing.
	Cases Used	Statistics for each analysis are based on cases with no missing data for any variable in the analysis.
Syntax		ONEWAY Inhibition BY Salt /STATISTICS DESCRIPTIVES EFFECTS /MISSING ANALYSIS /POSTHOC=DUNCAN LSD ALPHA(0.05).
Resources	Processor Time	00:00:00.02
	Elapsed Time	00:00:00.06

```
[DataSet0]
```

Descriptives

Inhibition

		N	Mean	Std. Deviation	Std. Error	95% Confidence ...
						Lower Bound
0.0		2	28.6700	.00000	.00000	28.6700
0.5		2	32.6700	.82024	.58000	25.3004
1.0		2	23.3300	1.41421	1.00000	10.6238
1.5		2	18.0000	.82024	.58000	10.6304
2.0		2	18.6700	.82024	.58000	11.3004
2.5		2	12.3300	.00000	.00000	12.3300
3.0		2	.0000	.00000	.00000	.0000
Total		14	19.0957	10.43419	2.78865	13.0712
Model	Fixed Effects			.75767	.20249	18.6169
	Random Effects				4.09896	9.0659

Descriptives

Inhibition

		95% Confidence Interval for Mean	Minimum	Maximum	Between-Component Variance
		Upper Bound			
0.0		28.6700	28.67	28.67	
0.5		40.0396	32.09	33.25	
1.0		36.0362	22.33	24.33	
1.5		25.3696	17.42	18.58	
2.0		26.0396	18.09	19.25	
2.5		12.3300	12.33	12.33	
3.0		.0000	.00	.00	
Total		25.1202	.00	33.25	
Model	Fixed Effects	19.5745			
	Random Effects	29.1255			117.32303

ANOVA

Inhibition

	Sum of Squares	df	Mean Square	F	Sig.
Between Groups	1411.321	6	235.220	409.750	.000
Within Groups	4.018	7	.574		
Total	1415.339	13			

Post Hoc Tests

Multiple Comparisons

Dependent Variable: Inhibition

	(I) Salt	(J) Salt	Mean Difference (I-J)	Std. Error	Sig.	95% Confidence Interval	
						Lower Bound	Upper Bound
LSD	0.0	0.5	-4.00000*	.75767	.001	-5.7916	-2.2084
		1.0	5.34000*	.75767	.000	3.5484	7.1316
		1.5	10.67000*	.75767	.000	8.8784	12.4616
		2.0	10.00000*	.75767	.000	8.2084	11.7916
		2.5	16.34000*	.75767	.000	14.5484	18.1316
		3.0	28.67000*	.75767	.000	26.8784	30.4616
	0.5	0.0	4.00000*	.75767	.001	2.2084	5.7916
		1.0	9.34000*	.75767	.000	7.5484	11.1316
		1.5	14.67000*	.75767	.000	12.8784	16.4616
		2.0	14.00000*	.75767	.000	12.2084	15.7916
		2.5	20.34000*	.75767	.000	18.5484	22.1316
		3.0	32.67000*	.75767	.000	30.8784	34.4616
	1.0	0.0	-5.34000*	.75767	.000	-7.1316	-3.5484
		0.5	-9.34000*	.75767	.000	-11.1316	-7.5484
		1.5	5.33000*	.75767	.000	3.5384	7.1216
		2.0	4.66000*	.75767	.000	2.8684	6.4516
		2.5	11.00000*	.75767	.000	9.2084	12.7916
		3.0	23.33000*	.75767	.000	21.5384	25.1216
	1.5	0.0	-10.67000*	.75767	.000	-12.4616	-8.8784
		0.5	-14.67000*	.75767	.000	-16.4616	-12.8784
		1.0	-5.33000*	.75767	.000	-7.1216	-3.5384
		2.0	-.67000	.75767	.406	-2.4616	1.1216
		2.5	5.67000*	.75767	.000	3.8784	7.4616
		3.0	18.00000*	.75767	.000	16.2084	19.7916
	2.0	0.0	-10.00000*	.75767	.000	-11.7916	-8.2084
		0.5	-14.00000*	.75767	.000	-15.7916	-12.2084
		1.0	-4.66000*	.75767	.000	-6.4516	-2.8684
		1.5	.67000	.75767	.406	-1.1216	2.4616
		2.5	6.34000*	.75767	.000	4.5484	8.1316
		3.0	18.67000*	.75767	.000	16.8784	20.4616
	2.5	0.0	-16.34000*	.75767	.000	-18.1316	-14.5484
		0.5	-20.34000*	.75767	.000	-22.1316	-18.5484
		1.0	-11.00000*	.75767	.000	-12.7916	-9.2084
		1.5	-5.67000*	.75767	.000	-7.4616	-3.8784
		2.0	-6.34000*	.75767	.000	-8.1316	-4.5484
		3.0	12.33000*	.75767	.000	10.5384	14.1216

Multiple Comparisons

Dependent Variable: Inhibition

(I) Salt	(J) Salt	Mean Difference (I-J)	Std. Error	Sig.	95% Confidence Interval	
					Lower Bound	Upper Bound
3.0	0.0	-28.67000*	.75767	.000	-30.4616	-26.8784
	0.5	-32.67000*	.75767	.000	-34.4616	-30.8784
	1.0	-23.33000*	.75767	.000	-25.1216	-21.5384
	1.5	-18.00000*	.75767	.000	-19.7916	-16.2084
	2.0	-18.67000*	.75767	.000	-20.4616	-16.8784
	2.5	-12.33000*	.75767	.000	-14.1216	-10.5384

*. The mean difference is significant at the 0.05 level.

Homogeneous Subsets

Inhibition

	Salt	N	Subset for alpha = 0.05					
			1	2	3	4	5	6
Duncan[a]	3.0	2	.0000					
	2.5	2		12.3300				
	1.5	2			18.0000			
	2.0	2			18.6700			
	1.0	2				23.3300		
	0.0	2					28.6700	
	0.5	2						32.6700
	Sig.		1.000	1.000	.406	1.000	1.000	1.000

Means for groups in homogeneous subsets are displayed.

a. Uses Harmonic Mean Sample Size = 2.000.
