# Supplementary material for: Biological characteristics of marine Streptomyces SK3 and optimization of cultivation conditions for production of compounds against Vibiriosis pathogen isolated from cultured white shrimp (Litopenaeus vannamei)
Source: PeerJ. 2024 Sep 24;12:e18053. doi: 10.7717/peerj.18053 (PMC11430173; doi:10.7717/peerj.18053)
Supplement: Supplemental Information 12 — Raw data exported from the statistical software SPSS (version 22) was analyzed using one-way ANOVA at a 95% confidence interval (p < 0.05) of incubation temperature. [file peerj-12-18053-s012.pdf]

```
ONEWAY Inhibition BY Temperature
  /STATISTICS DESCRIPTIVES EFFECTS
  /MISSING ANALYSIS
  /POSTHOC=DUNCAN LSD ALPHA(0.05).
```

Oneway

Notes

Output Created		27-APR-2024 12:33:16
Comments		
Input	Active Dataset	DataSet0
	Filter	<none>
	Weight	<none>
	Split File	<none>
	N of Rows in Working Data File	10
Missing Value Handling	Definition of Missing	User-defined missing values are treated as missing.
	Cases Used	Statistics for each analysis are based on cases with no missing data for any variable in the analysis.
Syntax		ONEWAY Inhibition BY Temperature /STATISTICS DESCRIPTIVES EFFECTS /MISSING ANALYSIS /POSTHOC=DUNCAN LSD ALPHA(0.05).
Resources	Processor Time	00:00:00.02
	Elapsed Time	00:00:00.03

```
[DataSet0]
```

Descriptives

Inhibition

		N	Mean	Std. Deviation	Std. Error	95% Confidence ... Lower Bound
10		2	.0000	.00000	.00000	.0000
20		2	16.3150	.82731	.58500	8.8819
30		2	28.0000	.00000	.00000	28.0000
40		2	12.3200	.82024	.58000	4.9504
50		2	12.3300	.83439	.59000	4.8333
Total		10	13.7930	9.47257	2.99549	7.0167
Model	Fixed Effects			.64085	.20265	13.2721
	Random Effects				4.48752	1.3337

Descriptives

Inhibition

		95% Confidence Interval for Mean Upper Bound	Minimum	Maximum	Between-Component Variance
10		.0000	.00	.00	
20		23.7481	15.73	16.90	
30		28.0000	28.00	28.00	
40		19.6896	11.74	12.90	
50		19.8267	11.74	12.92	
Total		20.5693	.00	28.00	
Model	Fixed Effects	14.3139			
	Random Effects	26.2523			100.48373

ANOVA

Inhibition

	Sum of Squares	df	Mean Square	F	Sig.
Between Groups	805.513	4	201.378	490.341	.000
Within Groups	2.053	5	.411		
Total	807.566	9			

Post Hoc Tests

Multiple Comparisons

Dependent Variable: Inhibition

	(I) Temperature	(J) Temperature	Mean Difference (I-J)	Std. Error	Sig.	95% ... Lower Bound
LSD	10	20	-16.31500*	.64085	.000	-17.9624
		30	-28.00000*	.64085	.000	-29.6474
		40	-12.32000*	.64085	.000	-13.9674
		50	-12.33000*	.64085	.000	-13.9774
	20	10	16.31500*	.64085	.000	14.6676
		30	-11.68500*	.64085	.000	-13.3324
		40	3.99500*	.64085	.002	2.3476
		50	3.98500*	.64085	.002	2.3376
	30	10	28.00000*	.64085	.000	26.3526
		20	11.68500*	.64085	.000	10.0376
		40	15.68000*	.64085	.000	14.0326
		50	15.67000*	.64085	.000	14.0226
	40	10	12.32000*	.64085	.000	10.6726
		20	-3.99500*	.64085	.002	-5.6424
		30	-15.68000*	.64085	.000	-17.3274
		50	-.01000	.64085	.988	-1.6574
	50	10	12.33000*	.64085	.000	10.6826
		20	-3.98500*	.64085	.002	-5.6324
		30	-15.67000*	.64085	.000	-17.3174
		40	.01000	.64085	.988	-1.6374

Multiple Comparisons

Dependent Variable: Inhibition

	(I) Temperature	(J) Temperature	95% Confidence Upper Bound
LSD	10	20	-14.6676
		30	-26.3526
		40	-10.6726
		50	-10.6826
	20	10	17.9624
		30	-10.0376
		40	5.6424
		50	5.6324
	30	10	29.6474
		20	13.3324
		40	17.3274
		50	17.3174
	40	10	13.9674
		20	-2.3476
		30	-14.0326
		50	1.6374
	50	10	13.9774
		20	-2.3376
		30	-14.0226
		40	1.6574

*. The mean difference is significant at the 0.05 level.

Homogeneous Subsets

Inhibition

	Temperature	N	Subset for alpha = 0.05			
			1	2	3	4
Duncan[a]	10	2	.0000			
	40	2		12.3200		
	50	2		12.3300		
	20	2			16.3150	
	30	2				28.0000
	Sig.		1.000	.988	1.000	1.000

Means for groups in homogeneous subsets are displayed.

a. Uses Harmonic Mean Sample Size = 2.000.
