# Supplementary material for: Biological characteristics of marine Streptomyces SK3 and optimization of cultivation conditions for production of compounds against Vibiriosis pathogen isolated from cultured white shrimp (Litopenaeus vannamei)
Source: PeerJ. 2024 Sep 24;12:e18053. doi: 10.7717/peerj.18053 (PMC11430173; doi:10.7717/peerj.18053)
Supplement: Supplemental Information 13 — Raw data exported from the statistical software SPSS (version 22) was analyzed using one-way ANOVA at a 95% confidence interval (p < 0.05) of protein secretion to liquid medium. [file peerj-12-18053-s013.pdf]

```
ONEWAY Protein BY Time
  /STATISTICS DESCRIPTIVES EFFECTS
  /MISSING ANALYSIS
  /POSTHOC=DUNCAN LSD ALPHA(0.05).
```

Oneway

Notes

Output Created		27-APR-2024 11:44:22
Comments		
Input	Active Dataset	DataSet0
	Filter	<none>
	Weight	<none>
	Split File	<none>
	N of Rows in Working Data File	36
Missing Value Handling	Definition of Missing	User-defined missing values are treated as missing.
	Cases Used	Statistics for each analysis are based on cases with no missing data for any variable in the analysis.
Syntax		ONEWAY Protein BY Time /STATISTICS DESCRIPTIVES EFFECTS /MISSING ANALYSIS /POSTHOC=DUNCAN LSD ALPHA(0.05).
Resources	Processor Time	00:00:00.13
	Elapsed Time	00:00:00.33

[DataSet0]

Descriptives

Protein

		N	Mean	Std. Deviation	Std. Error	95% Confidence ...
						Lower Bound
0		2	.00000	.000000	.000000	.00000
4		2	.19500	.007071	.005000	.13147
8		2	.30000	.007071	.005000	.23647
12		2	.39950	.003536	.002500	.36773
16		2	.49850	.000707	.000500	.49215
20		2	.60250	.002121	.001500	.58344
24		2	.72700	.001414	.001000	.71429
48		2	1.54500	.007071	.005000	1.48147
72		2	4.90250	.002121	.001500	4.88344
96		2	8.35550	.006364	.004500	8.29832
120		2	8.33550	.006364	.004500	8.27832
144		2	8.34050	.014849	.010500	8.20708
168		2	7.11050	.014849	.010500	6.97708
192		2	6.91050	.028991	.020500	6.65002
216		2	6.42050	.013435	.009500	6.29979
240		2	6.35600	.007071	.005000	6.29247
264		2	6.40050	.013435	.009500	6.27979
288		2	6.40250	.012021	.008500	6.29450
Total		36	4.10011	3.346699	.557783	2.96775
Model	Fixed Effects			.010781	.001797	4.09634
	Random Effects				.800339	2.41154

Descriptives

Protein

		95% Confidence Interval for Mean			Between-Component Variance
		Upper Bound	Minimum	Maximum	
0		.00000	.000	.000	
4		.25853	.190	.200	
8		.36353	.295	.305	
12		.43127	.397	.402	
16		.50485	.498	.499	
20		.62156	.601	.604	
24		.73971	.726	.728	
48		1.60853	1.540	1.550	
72		4.92156	4.901	4.904	
96		8.41268	8.351	8.360	
120		8.39268	8.331	8.340	
144		8.47392	8.330	8.351	
168		7.24392	7.100	7.121	
192		7.17098	6.890	6.931	
216		6.54121	6.411	6.430	
240		6.41953	6.351	6.361	
264		6.52121	6.391	6.410	
288		6.51050	6.394	6.411	
Total		5.23247	.000	8.360	
Model	Fixed Effects	4.10389			
	Random Effects	5.78868			11.529698

ANOVA

Protein

	Sum of Squares	df	Mean Square	F	Sig.
Between Groups	392.012	17	23.060	198408.809	.000
Within Groups	.002	18	.000		
Total	392.014	35			

Post Hoc Tests

Multiple Comparisons

Dependent Variable: Protein

	(I) Time	(J) Time	Mean Difference (I-J)	Std. Error	Sig.	95% Confidence Interval	
						Lower Bound	Upper Bound
LSD	0	4	-.195000*	.010781	.000	-.21765	-.17235
		8	-.300000*	.010781	.000	-.32265	-.27735
		12	-.399500*	.010781	.000	-.42215	-.37685
		16	-.498500*	.010781	.000	-.52115	-.47585
		20	-.602500*	.010781	.000	-.62515	-.57985
		24	-.727000*	.010781	.000	-.74965	-.70435
		48	-1.545000*	.010781	.000	-1.56765	-1.52235
		72	-4.902500*	.010781	.000	-4.92515	-4.87985
		96	-8.355500*	.010781	.000	-8.37815	-8.33285
		120	-8.335500*	.010781	.000	-8.35815	-8.31285
		144	-8.340500*	.010781	.000	-8.36315	-8.31785
		168	-7.110500*	.010781	.000	-7.13315	-7.08785
		192	-6.910500*	.010781	.000	-6.93315	-6.88785
		216	-6.420500*	.010781	.000	-6.44315	-6.39785
		240	-6.356000*	.010781	.000	-6.37865	-6.33335
		264	-6.400500*	.010781	.000	-6.42315	-6.37785
		288	-6.402500*	.010781	.000	-6.42515	-6.37985
	4	0	.195000*	.010781	.000	.17235	.21765
		8	-.105000*	.010781	.000	-.12765	-.08235
		12	-.204500*	.010781	.000	-.22715	-.18185
		16	-.303500*	.010781	.000	-.32615	-.28085
		20	-.407500*	.010781	.000	-.43015	-.38485
		24	-.532000*	.010781	.000	-.55465	-.50935
		48	-1.350000*	.010781	.000	-1.37265	-1.32735
		72	-4.707500*	.010781	.000	-4.73015	-4.68485
		96	-8.160500*	.010781	.000	-8.18315	-8.13785
		120	-8.140500*	.010781	.000	-8.16315	-8.11785
		144	-8.145500*	.010781	.000	-8.16815	-8.12285
		168	-6.915500*	.010781	.000	-6.93815	-6.89285
		192	-6.715500*	.010781	.000	-6.73815	-6.69285
		216	-6.225500*	.010781	.000	-6.24815	-6.20285
		240	-6.161000*	.010781	.000	-6.18365	-6.13835
		264	-6.205500*	.010781	.000	-6.22815	-6.18285
		288	-6.207500*	.010781	.000	-6.23015	-6.18485
	8	0	.300000*	.010781	.000	.27735	.32265
		4	.105000*	.010781	.000	.08235	.12765
		12	-.099500*	.010781	.000	-.12215	-.07685

Multiple Comparisons

Dependent Variable: Protein

(I) Time	(J) Time	Mean Difference (I-J)	Std. Error	Sig.	95% Confidence Interval Lower Bound	Upper Bound
	16	-.198500*	.010781	.000	-.22115	-.17585
	20	-.302500*	.010781	.000	-.32515	-.27985
	24	-.427000*	.010781	.000	-.44965	-.40435
	48	-1.245000*	.010781	.000	-1.26765	-1.22235
	72	-4.602500*	.010781	.000	-4.62515	-4.57985
	96	-8.055500*	.010781	.000	-8.07815	-8.03285
	120	-8.035500*	.010781	.000	-8.05815	-8.01285
	144	-8.040500*	.010781	.000	-8.06315	-8.01785
	168	-6.810500*	.010781	.000	-6.83315	-6.78785
	192	-6.610500*	.010781	.000	-6.63315	-6.58785
	216	-6.120500*	.010781	.000	-6.14315	-6.09785
	240	-6.056000*	.010781	.000	-6.07865	-6.03335
	264	-6.100500*	.010781	.000	-6.12315	-6.07785
	288	-6.102500*	.010781	.000	-6.12515	-6.07985
12	0	.399500*	.010781	.000	.37685	.42215
	4	.204500*	.010781	.000	.18185	.22715
	8	.099500*	.010781	.000	.07685	.12215
	16	-.099000*	.010781	.000	-.12165	-.07635
	20	-.203000*	.010781	.000	-.22565	-.18035
	24	-.327500*	.010781	.000	-.35015	-.30485
	48	-1.145500*	.010781	.000	-1.16815	-1.12285
	72	-4.503000*	.010781	.000	-4.52565	-4.48035
	96	-7.956000*	.010781	.000	-7.97865	-7.93335
	120	-7.936000*	.010781	.000	-7.95865	-7.91335
	144	-7.941000*	.010781	.000	-7.96365	-7.91835
	168	-6.711000*	.010781	.000	-6.73365	-6.68835
	192	-6.511000*	.010781	.000	-6.53365	-6.48835
	216	-6.021000*	.010781	.000	-6.04365	-5.99835
	240	-5.956500*	.010781	.000	-5.97915	-5.93385
	264	-6.001000*	.010781	.000	-6.02365	-5.97835
	288	-6.003000*	.010781	.000	-6.02565	-5.98035
16	0	.498500*	.010781	.000	.47585	.52115
	4	.303500*	.010781	.000	.28085	.32615
	8	.198500*	.010781	.000	.17585	.22115
	12	.099000*	.010781	.000	.07635	.12165
	20	-.104000*	.010781	.000	-.12665	-.08135
	24	-.228500*	.010781	.000	-.25115	-.20585

Multiple Comparisons

Dependent Variable: Protein

(I) Time	(J) Time	Mean Difference (I-J)	Std. Error	Sig.	95% Confidence Interval Lower Bound	Upper Bound
	48	-1.046500*	.010781	.000	-1.06915	-1.02385
	72	-4.404000*	.010781	.000	-4.42665	-4.38135
	96	-7.857000*	.010781	.000	-7.87965	-7.83435
	120	-7.837000*	.010781	.000	-7.85965	-7.81435
	144	-7.842000*	.010781	.000	-7.86465	-7.81935
	168	-6.612000*	.010781	.000	-6.63465	-6.58935
	192	-6.412000*	.010781	.000	-6.43465	-6.38935
	216	-5.922000*	.010781	.000	-5.94465	-5.89935
	240	-5.857500*	.010781	.000	-5.88015	-5.83485
	264	-5.902000*	.010781	.000	-5.92465	-5.87935
	288	-5.904000*	.010781	.000	-5.92665	-5.88135
20	0	.602500*	.010781	.000	.57985	.62515
	4	.407500*	.010781	.000	.38485	.43015
	8	.302500*	.010781	.000	.27985	.32515
	12	.203000*	.010781	.000	.18035	.22565
	16	.104000*	.010781	.000	.08135	.12665
	24	-.124500*	.010781	.000	-.14715	-.10185
	48	-.942500*	.010781	.000	-.96515	-.91985
	72	-4.300000*	.010781	.000	-4.32265	-4.27735
	96	-7.753000*	.010781	.000	-7.77565	-7.73035
	120	-7.733000*	.010781	.000	-7.75565	-7.71035
	144	-7.738000*	.010781	.000	-7.76065	-7.71535
	168	-6.508000*	.010781	.000	-6.53065	-6.48535
	192	-6.308000*	.010781	.000	-6.33065	-6.28535
	216	-5.818000*	.010781	.000	-5.84065	-5.79535
	240	-5.753500*	.010781	.000	-5.77615	-5.73085
	264	-5.798000*	.010781	.000	-5.82065	-5.77535
	288	-5.800000*	.010781	.000	-5.82265	-5.77735
24	0	.727000*	.010781	.000	.70435	.74965
	4	.532000*	.010781	.000	.50935	.55465
	8	.427000*	.010781	.000	.40435	.44965
	12	.327500*	.010781	.000	.30485	.35015
	16	.228500*	.010781	.000	.20585	.25115
	20	.124500*	.010781	.000	.10185	.14715
	48	-.818000*	.010781	.000	-.84065	-.79535
	72	-4.175500*	.010781	.000	-4.19815	-4.15285
	96	-7.628500*	.010781	.000	-7.65115	-7.60585

Multiple Comparisons

Dependent Variable: Protein

(I) Time	(J) Time	Mean Difference (I-J)	Std. Error	Sig.	95% Confidence Interval Lower Bound	Upper Bound
	120	-7.608500*	.010781	.000	-7.63115	-7.58585
	144	-7.613500*	.010781	.000	-7.63615	-7.59085
	168	-6.383500*	.010781	.000	-6.40615	-6.36085
	192	-6.183500*	.010781	.000	-6.20615	-6.16085
	216	-5.693500*	.010781	.000	-5.71615	-5.67085
	240	-5.629000*	.010781	.000	-5.65165	-5.60635
	264	-5.673500*	.010781	.000	-5.69615	-5.65085
	288	-5.675500*	.010781	.000	-5.69815	-5.65285
48	0	1.545000*	.010781	.000	1.52235	1.56765
	4	1.350000*	.010781	.000	1.32735	1.37265
	8	1.245000*	.010781	.000	1.22235	1.26765
	12	1.145500*	.010781	.000	1.12285	1.16815
	16	1.046500*	.010781	.000	1.02385	1.06915
	20	.942500*	.010781	.000	.91985	.96515
	24	.818000*	.010781	.000	.79535	.84065
	72	-3.357500*	.010781	.000	-3.38015	-3.33485
	96	-6.810500*	.010781	.000	-6.83315	-6.78785
	120	-6.790500*	.010781	.000	-6.81315	-6.76785
	144	-6.795500*	.010781	.000	-6.81815	-6.77285
	168	-5.565500*	.010781	.000	-5.58815	-5.54285
	192	-5.365500*	.010781	.000	-5.38815	-5.34285
	216	-4.875500*	.010781	.000	-4.89815	-4.85285
	240	-4.811000*	.010781	.000	-4.83365	-4.78835
	264	-4.855500*	.010781	.000	-4.87815	-4.83285
	288	-4.857500*	.010781	.000	-4.88015	-4.83485
72	0	4.902500*	.010781	.000	4.87985	4.92515
	4	4.707500*	.010781	.000	4.68485	4.73015
	8	4.602500*	.010781	.000	4.57985	4.62515
	12	4.503000*	.010781	.000	4.48035	4.52565
	16	4.404000*	.010781	.000	4.38135	4.42665
	20	4.300000*	.010781	.000	4.27735	4.32265
	24	4.175500*	.010781	.000	4.15285	4.19815
	48	3.357500*	.010781	.000	3.33485	3.38015
	96	-3.453000*	.010781	.000	-3.47565	-3.43035
	120	-3.433000*	.010781	.000	-3.45565	-3.41035
	144	-3.438000*	.010781	.000	-3.46065	-3.41535
	168	-2.208000*	.010781	.000	-2.23065	-2.18535

Multiple Comparisons

Dependent Variable: Protein

(I) Time	(J) Time	Mean Difference (I-J)	Std. Error	Sig.	95% Confidence Interval Lower Bound	Upper Bound
	192	-2.008000*	.010781	.000	-2.03065	-1.98535
	216	-1.518000*	.010781	.000	-1.54065	-1.49535
	240	-1.453500*	.010781	.000	-1.47615	-1.43085
	264	-1.498000*	.010781	.000	-1.52065	-1.47535
	288	-1.500000*	.010781	.000	-1.52265	-1.47735
96	0	8.355500*	.010781	.000	8.33285	8.37815
	4	8.160500*	.010781	.000	8.13785	8.18315
	8	8.055500*	.010781	.000	8.03285	8.07815
	12	7.956000*	.010781	.000	7.93335	7.97865
	16	7.857000*	.010781	.000	7.83435	7.87965
	20	7.753000*	.010781	.000	7.73035	7.77565
	24	7.628500*	.010781	.000	7.60585	7.65115
	48	6.810500*	.010781	.000	6.78785	6.83315
	72	3.453000*	.010781	.000	3.43035	3.47565
	120	.020000	.010781	.080	-.00265	.04265
	144	.015000	.010781	.181	-.00765	.03765
	168	1.245000*	.010781	.000	1.22235	1.26765
	192	1.445000*	.010781	.000	1.42235	1.46765
	216	1.935000*	.010781	.000	1.91235	1.95765
	240	1.999500*	.010781	.000	1.97685	2.02215
	264	1.955000*	.010781	.000	1.93235	1.97765
	288	1.953000*	.010781	.000	1.93035	1.97565
120	0	8.335500*	.010781	.000	8.31285	8.35815
	4	8.140500*	.010781	.000	8.11785	8.16315
	8	8.035500*	.010781	.000	8.01285	8.05815
	12	7.936000*	.010781	.000	7.91335	7.95865
	16	7.837000*	.010781	.000	7.81435	7.85965
	20	7.733000*	.010781	.000	7.71035	7.75565
	24	7.608500*	.010781	.000	7.58585	7.63115
	48	6.790500*	.010781	.000	6.76785	6.81315
	72	3.433000*	.010781	.000	3.41035	3.45565
	96	-.020000	.010781	.080	-.04265	.00265
	144	-.005000	.010781	.648	-.02765	.01765
	168	1.225000*	.010781	.000	1.20235	1.24765
	192	1.425000*	.010781	.000	1.40235	1.44765
	216	1.915000*	.010781	.000	1.89235	1.93765
	240	1.979500*	.010781	.000	1.95685	2.00215

Multiple Comparisons

Dependent Variable: Protein

(I) Time	(J) Time	Mean Difference (I-J)	Std. Error	Sig.	95% Confidence Interval Lower Bound	Upper Bound
	264	1.935000*	.010781	.000	1.91235	1.95765
	288	1.933000*	.010781	.000	1.91035	1.95565
144	0	8.340500*	.010781	.000	8.31785	8.36315
	4	8.145500*	.010781	.000	8.12285	8.16815
	8	8.040500*	.010781	.000	8.01785	8.06315
	12	7.941000*	.010781	.000	7.91835	7.96365
	16	7.842000*	.010781	.000	7.81935	7.86465
	20	7.738000*	.010781	.000	7.71535	7.76065
	24	7.613500*	.010781	.000	7.59085	7.63615
	48	6.795500*	.010781	.000	6.77285	6.81815
	72	3.438000*	.010781	.000	3.41535	3.46065
	96	-.015000	.010781	.181	-.03765	.00765
	120	.005000	.010781	.648	-.01765	.02765
	168	1.230000*	.010781	.000	1.20735	1.25265
	192	1.430000*	.010781	.000	1.40735	1.45265
	216	1.920000*	.010781	.000	1.89735	1.94265
	240	1.984500*	.010781	.000	1.96185	2.00715
	264	1.940000*	.010781	.000	1.91735	1.96265
	288	1.938000*	.010781	.000	1.91535	1.96065
168	0	7.110500*	.010781	.000	7.08785	7.13315
	4	6.915500*	.010781	.000	6.89285	6.93815
	8	6.810500*	.010781	.000	6.78785	6.83315
	12	6.711000*	.010781	.000	6.68835	6.73365
	16	6.612000*	.010781	.000	6.58935	6.63465
	20	6.508000*	.010781	.000	6.48535	6.53065
	24	6.383500*	.010781	.000	6.36085	6.40615
	48	5.565500*	.010781	.000	5.54285	5.58815
	72	2.208000*	.010781	.000	2.18535	2.23065
	96	-1.245000*	.010781	.000	-1.26765	-1.22235
	120	-1.225000*	.010781	.000	-1.24765	-1.20235
	144	-1.230000*	.010781	.000	-1.25265	-1.20735
	192	.200000*	.010781	.000	.17735	.22265
	216	.690000*	.010781	.000	.66735	.71265
	240	.754500*	.010781	.000	.73185	.77715
	264	.710000*	.010781	.000	.68735	.73265
	288	.708000*	.010781	.000	.68535	.73065

Multiple Comparisons

Dependent Variable: Protein

(I) Time	(J) Time	Mean Difference (I-J)	Std. Error	Sig.	95% Confidence Interval Lower Bound	Upper Bound
192	0	6.910500*	.010781	.000	6.88785	6.93315
	4	6.715500*	.010781	.000	6.69285	6.73815
	8	6.610500*	.010781	.000	6.58785	6.63315
	12	6.511000*	.010781	.000	6.48835	6.53365
	16	6.412000*	.010781	.000	6.38935	6.43465
	20	6.308000*	.010781	.000	6.28535	6.33065
	24	6.183500*	.010781	.000	6.16085	6.20615
	48	5.365500*	.010781	.000	5.34285	5.38815
	72	2.008000*	.010781	.000	1.98535	2.03065
	96	-1.445000*	.010781	.000	-1.46765	-1.42235
	120	-1.425000*	.010781	.000	-1.44765	-1.40235
	144	-1.430000*	.010781	.000	-1.45265	-1.40735
	168	-.200000*	.010781	.000	-.22265	-.17735
	216	.490000*	.010781	.000	.46735	.51265
	240	.554500*	.010781	.000	.53185	.57715
	264	.510000*	.010781	.000	.48735	.53265
	288	.508000*	.010781	.000	.48535	.53065
216	0	6.420500*	.010781	.000	6.39785	6.44315
	4	6.225500*	.010781	.000	6.20285	6.24815
	8	6.120500*	.010781	.000	6.09785	6.14315
	12	6.021000*	.010781	.000	5.99835	6.04365
	16	5.922000*	.010781	.000	5.89935	5.94465
	20	5.818000*	.010781	.000	5.79535	5.84065
	24	5.693500*	.010781	.000	5.67085	5.71615
	48	4.875500*	.010781	.000	4.85285	4.89815
	72	1.518000*	.010781	.000	1.49535	1.54065
	96	-1.935000*	.010781	.000	-1.95765	-1.91235
	120	-1.915000*	.010781	.000	-1.93765	-1.89235
	144	-1.920000*	.010781	.000	-1.94265	-1.89735
	168	-.690000*	.010781	.000	-.71265	-.66735
	192	-.490000*	.010781	.000	-.51265	-.46735
	240	.064500*	.010781	.000	.04185	.08715
	264	.020000	.010781	.080	-.00265	.04265
	288	.018000	.010781	.112	-.00465	.04065
240	0	6.356000*	.010781	.000	6.33335	6.37865
	4	6.161000*	.010781	.000	6.13835	6.18365
	8	6.056000*	.010781	.000	6.03335	6.07865

Multiple Comparisons

Dependent Variable: Protein

(I) Time	(J) Time	Mean Difference (I-J)	Std. Error	Sig.	95% Confidence Interval Lower Bound	Upper Bound
	12	5.956500*	.010781	.000	5.93385	5.97915
	16	5.857500*	.010781	.000	5.83485	5.88015
	20	5.753500*	.010781	.000	5.73085	5.77615
	24	5.629000*	.010781	.000	5.60635	5.65165
	48	4.811000*	.010781	.000	4.78835	4.83365
	72	1.453500*	.010781	.000	1.43085	1.47615
	96	-1.999500*	.010781	.000	-2.02215	-1.97685
	120	-1.979500*	.010781	.000	-2.00215	-1.95685
	144	-1.984500*	.010781	.000	-2.00715	-1.96185
	168	-.754500*	.010781	.000	-.77715	-.73185
	192	-.554500*	.010781	.000	-.57715	-.53185
	216	-.064500*	.010781	.000	-.08715	-.04185
	264	-.044500*	.010781	.001	-.06715	-.02185
	288	-.046500*	.010781	.000	-.06915	-.02385
264	0	6.400500*	.010781	.000	6.37785	6.42315
	4	6.205500*	.010781	.000	6.18285	6.22815
	8	6.100500*	.010781	.000	6.07785	6.12315
	12	6.001000*	.010781	.000	5.97835	6.02365
	16	5.902000*	.010781	.000	5.87935	5.92465
	20	5.798000*	.010781	.000	5.77535	5.82065
	24	5.673500*	.010781	.000	5.65085	5.69615
	48	4.855500*	.010781	.000	4.83285	4.87815
	72	1.498000*	.010781	.000	1.47535	1.52065
	96	-1.955000*	.010781	.000	-1.97765	-1.93235
	120	-1.935000*	.010781	.000	-1.95765	-1.91235
	144	-1.940000*	.010781	.000	-1.96265	-1.91735
	168	-.710000*	.010781	.000	-.73265	-.68735
	192	-.510000*	.010781	.000	-.53265	-.48735
	216	-.020000	.010781	.080	-.04265	.00265
	240	.044500*	.010781	.001	.02185	.06715
	288	-.002000	.010781	.855	-.02465	.02065
288	0	6.402500*	.010781	.000	6.37985	6.42515
	4	6.207500*	.010781	.000	6.18485	6.23015
	8	6.102500*	.010781	.000	6.07985	6.12515
	12	6.003000*	.010781	.000	5.98035	6.02565
	16	5.904000*	.010781	.000	5.88135	5.92665
	20	5.800000*	.010781	.000	5.77735	5.82265

Multiple Comparisons

Dependent Variable: Protein

(I) Time	(J) Time	Mean Difference (I-J)	Std. Error	Sig.	95% Confidence Interval Lower Bound	Upper Bound
	24	5.675500*	.010781	.000	5.65285	5.69815
	48	4.857500*	.010781	.000	4.83485	4.88015
	72	1.500000*	.010781	.000	1.47735	1.52265
	96	-1.953000*	.010781	.000	-1.97565	-1.93035
	120	-1.933000*	.010781	.000	-1.95565	-1.91035
	144	-1.938000*	.010781	.000	-1.96065	-1.91535
	168	-.708000*	.010781	.000	-.73065	-.68535
	192	-.508000*	.010781	.000	-.53065	-.48535
	216	-.018000	.010781	.112	-.04065	.00465
	240	.046500*	.010781	.000	.02385	.06915
	264	.002000	.010781	.855	-.02065	.02465

*. The mean difference is significant at the 0.05 level.

Homogeneous Subsets

Protein

	Time	N	Subset for alpha = 0.05 1	2	3	4	5	6
Duncan[a]	0	2	.00000					
	4	2		.19500				
	8	2			.30000			
	12	2				.39950		
	16	2					.49850	
	20	2						.60250
	24	2						
	48	2						
	72	2						
	240	2						
	264	2						
	288	2						
	216	2						
	192	2						
	168	2						
	120	2						
	144	2						
	96	2						
	Sig.		1.000	1.000	1.000	1.000	1.000	1.000

Protein

	Time	Subset for alpha = 0.05						
		7	8	9	10	11	12	13
Duncan[a]	0							
	4							
	8							
	12							
	16							
	20							
	24	.72700						
	48		1.54500					
	72			4.90250				
	240				6.35600			
	264					6.40050		
	288					6.40250		
	216					6.42050		
	192						6.91050	
	168							7.11050
	120							
	144							
	96							
	Sig.	1.000	1.000	1.000	1.000	.095	1.000	1.000

Protein

	Time	Subset for .
		14
Duncan[a]	0	
	4	
	8	
	12	
	16	
	20	
	24	
	48	
	72	
	240	
	264	
	288	
	216	
	192	
	168	
	120	8.33550
	144	8.34050
	96	8.35550
	Sig.	.095

Means for groups in homogeneous subsets are displayed.

a. Uses Harmonic Mean Sample Size = 2.000.
