# Supplementary material for: Biological characteristics of marine Streptomyces SK3 and optimization of cultivation conditions for production of compounds against Vibiriosis pathogen isolated from cultured white shrimp (Litopenaeus vannamei)
Source: PeerJ. 2024 Sep 24;12:e18053. doi: 10.7717/peerj.18053 (PMC11430173; doi:10.7717/peerj.18053)
Supplement: Supplemental Information 14 — Raw data exported from the statistical software SPSS (version 22) was analyzed using one-way ANOVA at a 95% confidence interval (p < 0.05) of trace elements. [file peerj-12-18053-s014.pdf]

```
ONEWAY Inhibition BY Trace
  /STATISTICS DESCRIPTIVES EFFECTS
  /MISSING ANALYSIS
  /POSTHOC=DUNCAN LSD ALPHA(0.05).
```

# Oneway

**Notes**

| | | |
|---|---|---|
| Output Created | | 27-APR-2024 13:48:51 |
| Comments | | |
| Input | Active Dataset | DataSet0 |
| | Filter | <none> |
| | Weight | <none> |
| | Split File | <none> |
| | N of Rows in Working Data File | 16 |
| Missing Value Handling | Definition of Missing | User-defined missing values are treated as missing. |
| | Cases Used | Statistics for each analysis are based on cases with no missing data for any variable in the analysis. |
| Syntax | | ONEWAY Inhibition BY Trace /STATISTICS DESCRIPTIVES EFFECTS /MISSING ANALYSIS /POSTHOC=DUNCAN LSD ALPHA(0.05). |
| Resources | Processor Time | 00:00:00.02 |
| | Elapsed Time | 00:00:00.06 |

[DataSet0]

**Descriptives**

Inhibition

| | | N | Mean | Std. Deviation | Std. Error | 95% Confidence ... |
|---|---|---|---|---|---|---|
| | | | | | | Lower Bound |
| FeSO4 | | 2 | .0000 | .00000 | .00000 | .0000 |
| MgSO4 | | 2 | 33.6700 | 1.41421 | 1.00000 | 20.9638 |
| CaCO3 | | 2 | 33.6700 | .82024 | .58000 | 26.3004 |
| K2HPO4 | | 2 | .0000 | .00000 | .00000 | .0000 |
| KH2PO4 | | 2 | 19.0000 | .82024 | .58000 | 11.6304 |
| KCl | | 2 | .0000 | .00000 | .00000 | .0000 |
| KNO3 | | 2 | .0000 | .00000 | .00000 | .0000 |
| No supplement | | 2 | 33.6700 | .82024 | .58000 | 26.3004 |
| Total | | 16 | 15.0013 | 16.18112 | 4.04528 | 6.3789 |
| Model | Fixed Effects | | | .70873 | .17718 | 14.5927 |
| | Random Effects | | | | 5.91865 | 1.0059 |

**Descriptives**

Inhibition

| | | 95% Confidence Interval for Mean | Minimum | Maximum | Between-Component Variance |
|---|---|---|---|---|---|
| | | Upper Bound | | | |
| FeSO4 | | .0000 | .00 | .00 | |
| MgSO4 | | 46.3762 | 32.67 | 34.67 | |
| CaCO3 | | 41.0396 | 33.09 | 34.25 | |
| K2HPO4 | | .0000 | .00 | .00 | |
| KH2PO4 | | 26.3696 | 18.42 | 19.58 | |
| KCl | | .0000 | .00 | .00 | |
| KNO3 | | .0000 | .00 | .00 | |
| No supplement | | 41.0396 | 33.09 | 34.25 | |
| Total | | 23.6236 | .00 | 34.67 | |
| Model | Fixed Effects | 15.4098 | | | |
| | Random Effects | 28.9966 | | | 279.99266 |

**ANOVA**

Inhibition

| | Sum of Squares | df | Mean Square | F | Sig. |
|---|---|---|---|---|---|
| Between Groups | 3923.413 | 7 | 560.488 | 1115.842 | .000 |
| Within Groups | 4.018 | 8 | .502 | | |
| Total | 3927.432 | 15 | | | |

# Post Hoc Tests

**Multiple Comparisons**

Dependent Variable: Inhibition

| | (I) Trace | (J) Trace | Mean Difference (I-J) | Std. Error | Sig. | 95% ... |
|---|---|---|---|---|---|---|
| | | | | | | Lower Bound |
| LSD | FeSO4 | MgSO4 | -33.67000* | .70873 | .000 | -35.3043 |
| | | CaCO3 | -33.67000* | .70873 | .000 | -35.3043 |
| | | K2HPO4 | .00000 | .70873 | 1.000 | -1.6343 |
| | | KH2PO4 | -19.00000* | .70873 | .000 | -20.6343 |
| | | KCl | .00000 | .70873 | 1.000 | -1.6343 |
| | | KNO3 | .00000 | .70873 | 1.000 | -1.6343 |
| | | No supplement | -33.67000* | .70873 | .000 | -35.3043 |
| | MgSO4 | FeSO4 | 33.67000* | .70873 | .000 | 32.0357 |
| | | CaCO3 | .00000 | .70873 | 1.000 | -1.6343 |
| | | K2HPO4 | 33.67000* | .70873 | .000 | 32.0357 |
| | | KH2PO4 | 14.67000* | .70873 | .000 | 13.0357 |
| | | KCl | 33.67000* | .70873 | .000 | 32.0357 |
| | | KNO3 | 33.67000* | .70873 | .000 | 32.0357 |
| | | No supplement | .00000 | .70873 | 1.000 | -1.6343 |
| | CaCO3 | FeSO4 | 33.67000* | .70873 | .000 | 32.0357 |
| | | MgSO4 | .00000 | .70873 | 1.000 | -1.6343 |
| | | K2HPO4 | 33.67000* | .70873 | .000 | 32.0357 |
| | | KH2PO4 | 14.67000* | .70873 | .000 | 13.0357 |
| | | KCl | 33.67000* | .70873 | .000 | 32.0357 |
| | | KNO3 | 33.67000* | .70873 | .000 | 32.0357 |
| | | No supplement | .00000 | .70873 | 1.000 | -1.6343 |
| | K2HPO4 | FeSO4 | .00000 | .70873 | 1.000 | -1.6343 |
| | | MgSO4 | -33.67000* | .70873 | .000 | -35.3043 |
| | | CaCO3 | -33.67000* | .70873 | .000 | -35.3043 |
| | | KH2PO4 | -19.00000* | .70873 | .000 | -20.6343 |
| | | KCl | .00000 | .70873 | 1.000 | -1.6343 |
| | | KNO3 | .00000 | .70873 | 1.000 | -1.6343 |
| | | No supplement | -33.67000* | .70873 | .000 | -35.3043 |
| | KH2PO4 | FeSO4 | 19.00000* | .70873 | .000 | 17.3657 |
| | | MgSO4 | -14.67000* | .70873 | .000 | -16.3043 |
| | | CaCO3 | -14.67000* | .70873 | .000 | -16.3043 |
| | | K2HPO4 | 19.00000* | .70873 | .000 | 17.3657 |
| | | KCl | 19.00000* | .70873 | .000 | 17.3657 |
| | | KNO3 | 19.00000* | .70873 | .000 | 17.3657 |
| | | No supplement | -14.67000* | .70873 | .000 | -16.3043 |

**Multiple Comparisons**

Dependent Variable: Inhibition

| | (I) Trace | (J) Trace | 95% Confidence Upper Bound |
|---|---|---|---|
| LSD | FeSO4 | MgSO4 | -32.0357 |
| | | CaCO3 | -32.0357 |
| | | K2HPO4 | 1.6343 |
| | | KH2PO4 | -17.3657 |
| | | KCl | 1.6343 |
| | | KNO3 | 1.6343 |
| | | No supplement | -32.0357 |
| | MgSO4 | FeSO4 | 35.3043 |
| | | CaCO3 | 1.6343 |
| | | K2HPO4 | 35.3043 |
| | | KH2PO4 | 16.3043 |
| | | KCl | 35.3043 |
| | | KNO3 | 35.3043 |
| | | No supplement | 1.6343 |
| | CaCO3 | FeSO4 | 35.3043 |
| | | MgSO4 | 1.6343 |
| | | K2HPO4 | 35.3043 |
| | | KH2PO4 | 16.3043 |
| | | KCl | 35.3043 |
| | | KNO3 | 35.3043 |
| | | No supplement | 1.6343 |
| | K2HPO4 | FeSO4 | 1.6343 |
| | | MgSO4 | -32.0357 |
| | | CaCO3 | -32.0357 |
| | | KH2PO4 | -17.3657 |
| | | KCl | 1.6343 |
| | | KNO3 | 1.6343 |
| | | No supplement | -32.0357 |
| | KH2PO4 | FeSO4 | 20.6343 |
| | | MgSO4 | -13.0357 |
| | | CaCO3 | -13.0357 |
| | | K2HPO4 | 20.6343 |
| | | KCl | 20.6343 |
| | | KNO3 | 20.6343 |
| | | No supplement | -13.0357 |

**Multiple Comparisons**

Dependent Variable: Inhibition

| (I) Trace | (J) Trace | Mean Difference (I-J) | Std. Error | Sig. | 95% ... Lower Bound |
|---|---|---|---|---|---|
| KCl | FeSO4 | .00000 | .70873 | 1.000 | -1.6343 |
| | MgSO4 | -33.67000* | .70873 | .000 | -35.3043 |
| | CaCO3 | -33.67000* | .70873 | .000 | -35.3043 |
| | K2HPO4 | .00000 | .70873 | 1.000 | -1.6343 |
| | KH2PO4 | -19.00000* | .70873 | .000 | -20.6343 |
| | KNO3 | .00000 | .70873 | 1.000 | -1.6343 |
| | No supplement | -33.67000* | .70873 | .000 | -35.3043 |
| KNO3 | FeSO4 | .00000 | .70873 | 1.000 | -1.6343 |
| | MgSO4 | -33.67000* | .70873 | .000 | -35.3043 |
| | CaCO3 | -33.67000* | .70873 | .000 | -35.3043 |
| | K2HPO4 | .00000 | .70873 | 1.000 | -1.6343 |
| | KH2PO4 | -19.00000* | .70873 | .000 | -20.6343 |
| | KCl | .00000 | .70873 | 1.000 | -1.6343 |
| | No supplement | -33.67000* | .70873 | .000 | -35.3043 |
| No supplement | FeSO4 | 33.67000* | .70873 | .000 | 32.0357 |
| | MgSO4 | .00000 | .70873 | 1.000 | -1.6343 |
| | CaCO3 | .00000 | .70873 | 1.000 | -1.6343 |
| | K2HPO4 | 33.67000* | .70873 | .000 | 32.0357 |
| | KH2PO4 | 14.67000* | .70873 | .000 | 13.0357 |
| | KCl | 33.67000* | .70873 | .000 | 32.0357 |
| | KNO3 | 33.67000* | .70873 | .000 | 32.0357 |

**Multiple Comparisons**

Dependent Variable: Inhibition

| (I) Trace | (J) Trace | 95% Confidence Upper Bound |
|---|---|---|
| KCl | FeSO4 | 1.6343 |
| | MgSO4 | -32.0357 |
| | CaCO3 | -32.0357 |
| | K2HPO4 | 1.6343 |
| | KH2PO4 | -17.3657 |
| | KNO3 | 1.6343 |
| | No supplement | -32.0357 |
| KNO3 | FeSO4 | 1.6343 |
| | MgSO4 | -32.0357 |
| | CaCO3 | -32.0357 |
| | K2HPO4 | 1.6343 |
| | KH2PO4 | -17.3657 |
| | KCl | 1.6343 |
| | No supplement | -32.0357 |
| No supplement | FeSO4 | 35.3043 |
| | MgSO4 | 1.6343 |
| | CaCO3 | 1.6343 |
| | K2HPO4 | 35.3043 |
| | KH2PO4 | 16.3043 |
| | KCl | 35.3043 |
| | KNO3 | 35.3043 |

*. The mean difference is significant at the 0.05 level.

## Homogeneous Subsets

**Inhibition**

| | Trace | N | Subset for alpha = 0.05 | | |
|---|---|---|---|---|---|
| | | | 1 | 2 | 3 |
| Duncan[a] | FeSO4 | 2 | .0000 | | |
| | K2HPO4 | 2 | .0000 | | |
| | KCl | 2 | .0000 | | |
| | KNO3 | 2 | .0000 | | |
| | KH2PO4 | 2 | | 19.0000 | |
| | MgSO4 | 2 | | | 33.6700 |
| | CaCO3 | 2 | | | 33.6700 |
| | No supplement | 2 | | | 33.6700 |
| | Sig. | | 1.000 | 1.000 | 1.000 |

Means for groups in homogeneous subsets are displayed.

a. Uses Harmonic Mean Sample Size = 2.000.
