# Supplementary material for: Biological characteristics of marine Streptomyces SK3 and optimization of cultivation conditions for production of compounds against Vibiriosis pathogen isolated from cultured white shrimp (Litopenaeus vannamei)
Source: PeerJ. 2024 Sep 24;12:e18053. doi: 10.7717/peerj.18053 (PMC11430173; doi:10.7717/peerj.18053)
Supplement: Supplemental Information 15 — Raw data exported from the statistical software SPSS (version 22) was analyzed using one-way ANOVA at a 95% confidence interval (p < 0.05) of type of medium. [file peerj-12-18053-s015.pdf]

```
ONEWAY Inhibition BY Medium1
  /STATISTICS DESCRIPTIVES EFFECTS
  /MISSING ANALYSIS
  /POSTHOC=DUNCAN LSD ALPHA(0.05).
```

Oneway

Notes

Output Created		03-MAY-2024 11:09:39
Comments		
Input	Active Dataset	DataSet0
	Filter	<none>
	Weight	<none>
	Split File	<none>
	N of Rows in Working Data File	18
Missing Value Handling	Definition of Missing	User-defined missing values are treated as missing.
	Cases Used	Statistics for each analysis are based on cases with no missing data for any variable in the analysis.
Syntax		ONEWAY Inhibition BY Medium1 /STATISTICS DESCRIPTIVES EFFECTS /MISSING ANALYSIS /POSTHOC=DUNCAN LSD ALPHA(0.05).
Resources	Processor Time	00:00:00.03
	Elapsed Time	00:00:00.09

```
[DataSet0]
```

Descriptives

Inhibition

		N	Mean	Std. Deviation	Std. Error	95% Confidence ...
						Lower Bound
YMB		2	30.0800	.82024	.58000	22.7104
TSB		2	21.0800	.82024	.58000	13.7104
MB		2	17.0800	.82024	.58000	9.7104
MHB		2	.0000	.00000	.00000	.0000
NB		2	.0000	.00000	.00000	.0000
ISP3		2	25.0000	.00000	.00000	25.0000
ISP4		2	16.0000	.00000	.00000	16.0000
ISP5		2	23.0800	.82024	.58000	15.7104
ISP7		2	20.0300	.04243	.03000	19.6488
Total		18	16.9278	10.16445	2.39578	11.8731
Model	Fixed Effects			.54701	.12893	16.6361
	Random Effects				3.48975	8.8804

Descriptives

Inhibition

		95% Confidence Interval for Mean	Minimum	Maximum	Between-Component Variance
		Upper Bound			
YMB		37.4496	29.50	30.66	
TSB		28.4496	20.50	21.66	
MB		24.4496	16.50	17.66	
MHB		.0000	.00	.00	
NB		.0000	.00	.00	
ISP3		25.0000	25.00	25.00	
ISP4		16.0000	16.00	16.00	
ISP5		30.4496	22.50	23.66	
ISP7		20.4112	20.00	20.06	
Total		21.9824	.00	30.66	
Model	Fixed Effects	17.2194			
	Random Effects	24.9751			109.45533

ANOVA

Inhibition

	Sum of Squares	df	Mean Square	F	Sig.
Between Groups	1753.679	8	219.210	732.599	.000
Within Groups	2.693	9	.299		
Total	1756.372	17			

Post Hoc Tests

Multiple Comparisons

Dependent Variable: Inhibition

	(I) Medium1	(J) Medium1	Mean Difference (I-J)	Std. Error	Sig.	95% ... Lower Bound
LSD	YMB	TSB	9.00000*	.54701	.000	7.7626
		MB	13.00000*	.54701	.000	11.7626
		MHB	30.08000*	.54701	.000	28.8426
		NB	30.08000*	.54701	.000	28.8426
		ISP3	5.08000*	.54701	.000	3.8426
		ISP4	14.08000*	.54701	.000	12.8426
		ISP5	7.00000*	.54701	.000	5.7626
		ISP7	10.05000*	.54701	.000	8.8126
	TSB	YMB	-9.00000*	.54701	.000	-10.2374
		MB	4.00000*	.54701	.000	2.7626
		MHB	21.08000*	.54701	.000	19.8426
		NB	21.08000*	.54701	.000	19.8426
		ISP3	-3.92000*	.54701	.000	-5.1574
		ISP4	5.08000*	.54701	.000	3.8426
		ISP5	-2.00000*	.54701	.005	-3.2374
		ISP7	1.05000	.54701	.087	-.1874
	MB	YMB	-13.00000*	.54701	.000	-14.2374
		TSB	-4.00000*	.54701	.000	-5.2374
		MHB	17.08000*	.54701	.000	15.8426
		NB	17.08000*	.54701	.000	15.8426
		ISP3	-7.92000*	.54701	.000	-9.1574
		ISP4	1.08000	.54701	.080	-.1574
		ISP5	-6.00000*	.54701	.000	-7.2374
		ISP7	-2.95000*	.54701	.000	-4.1874
	MHB	YMB	-30.08000*	.54701	.000	-31.3174
		TSB	-21.08000*	.54701	.000	-22.3174
		MB	-17.08000*	.54701	.000	-18.3174

Multiple Comparisons

Dependent Variable: Inhibition

	(I) Medium1	(J) Medium1	95% Confidence Upper Bound
LSD	YMB	TSB	10.2374
		MB	14.2374
		MHB	31.3174
		NB	31.3174
		ISP3	6.3174
		ISP4	15.3174
		ISP5	8.2374
		ISP7	11.2874
	TSB	YMB	-7.7626
		MB	5.2374
		MHB	22.3174
		NB	22.3174
		ISP3	-2.6826
		ISP4	6.3174
		ISP5	-.7626
		ISP7	2.2874
	MB	YMB	-11.7626
		TSB	-2.7626
		MHB	18.3174
		NB	18.3174
		ISP3	-6.6826
		ISP4	2.3174
		ISP5	-4.7626
		ISP7	-1.7126
	MHB	YMB	-28.8426
		TSB	-19.8426
		MB	-15.8426

Multiple Comparisons

Dependent Variable: Inhibition

(I) Medium1	(J) Medium1	Mean Difference (I-J)	Std. Error	Sig.	95% ... Lower Bound
	NB	.00000	.54701	1.000	-1.2374
	ISP3	-25.00000*	.54701	.000	-26.2374
	ISP4	-16.00000*	.54701	.000	-17.2374
	ISP5	-23.08000*	.54701	.000	-24.3174
	ISP7	-20.03000*	.54701	.000	-21.2674
NB	YMB	-30.08000*	.54701	.000	-31.3174
	TSB	-21.08000*	.54701	.000	-22.3174
	MB	-17.08000*	.54701	.000	-18.3174
	MHB	.00000	.54701	1.000	-1.2374
	ISP3	-25.00000*	.54701	.000	-26.2374
	ISP4	-16.00000*	.54701	.000	-17.2374
	ISP5	-23.08000*	.54701	.000	-24.3174
	ISP7	-20.03000*	.54701	.000	-21.2674
ISP3	YMB	-5.08000*	.54701	.000	-6.3174
	TSB	3.92000*	.54701	.000	2.6826
	MB	7.92000*	.54701	.000	6.6826
	MHB	25.00000*	.54701	.000	23.7626
	NB	25.00000*	.54701	.000	23.7626
	ISP4	9.00000*	.54701	.000	7.7626
	ISP5	1.92000*	.54701	.007	.6826
	ISP7	4.97000*	.54701	.000	3.7326
ISP4	YMB	-14.08000*	.54701	.000	-15.3174
	TSB	-5.08000*	.54701	.000	-6.3174
	MB	-1.08000	.54701	.080	-2.3174
	MHB	16.00000*	.54701	.000	14.7626
	NB	16.00000*	.54701	.000	14.7626
	ISP3	-9.00000*	.54701	.000	-10.2374
	ISP5	-7.08000*	.54701	.000	-8.3174
	ISP7	-4.03000*	.54701	.000	-5.2674
ISP5	YMB	-7.00000*	.54701	.000	-8.2374
	TSB	2.00000*	.54701	.005	.7626
	MB	6.00000*	.54701	.000	4.7626
	MHB	23.08000*	.54701	.000	21.8426
	NB	23.08000*	.54701	.000	21.8426
	ISP3	-1.92000*	.54701	.007	-3.1574
	ISP4	7.08000*	.54701	.000	5.8426
	ISP7	3.05000*	.54701	.000	1.8126

Multiple Comparisons

Dependent Variable: Inhibition

(I) Medium1	(J) Medium1	95% Confidence Upper Bound
	NB	1.2374
	ISP3	-23.7626
	ISP4	-14.7626
	ISP5	-21.8426
	ISP7	-18.7926
NB	YMB	-28.8426
	TSB	-19.8426
	MB	-15.8426
	MHB	1.2374
	ISP3	-23.7626
	ISP4	-14.7626
	ISP5	-21.8426
	ISP7	-18.7926
ISP3	YMB	-3.8426
	TSB	5.1574
	MB	9.1574
	MHB	26.2374
	NB	26.2374
	ISP4	10.2374
	ISP5	3.1574
	ISP7	6.2074
ISP4	YMB	-12.8426
	TSB	-3.8426
	MB	.1574
	MHB	17.2374
	NB	17.2374
	ISP3	-7.7626
	ISP5	-5.8426
	ISP7	-2.7926
ISP5	YMB	-5.7626
	TSB	3.2374
	MB	7.2374
	MHB	24.3174
	NB	24.3174
	ISP3	-.6826
	ISP4	8.3174
	ISP7	4.2874

Multiple Comparisons

Dependent Variable: Inhibition

(I) Medium1	(J) Medium1	Mean Difference (I-J)	Std. Error	Sig.	95% ... Lower Bound
ISP7	YMB	-10.05000*	.54701	.000	-11.2874
	TSB	-1.05000	.54701	.087	-2.2874
	MB	2.95000*	.54701	.000	1.7126
	MHB	20.03000*	.54701	.000	18.7926
	NB	20.03000*	.54701	.000	18.7926
	ISP3	-4.97000*	.54701	.000	-6.2074
	ISP4	4.03000*	.54701	.000	2.7926
	ISP5	-3.05000*	.54701	.000	-4.2874

Multiple Comparisons

Dependent Variable: Inhibition

(I) Medium1	(J) Medium1	95% Confidence Upper Bound
ISP7	YMB	-8.8126
	TSB	.1874
	MB	4.1874
	MHB	21.2674
	NB	21.2674
	ISP3	-3.7326
	ISP4	5.2674
	ISP5	-1.8126

*. The mean difference is significant at the 0.05 level.

Homogeneous Subsets

Inhibition

	Medium1	N	Subset for alpha = 0.05					
			1	2	3	4	5	6
Duncan[a]	MHB	2	.0000					
	NB	2	.0000					
	ISP4	2		16.0000				
	MB	2		17.0800				
	ISP7	2			20.0300			
	TSB	2			21.0800			
	ISP5	2				23.0800		
	ISP3	2					25.0000	
	YMB	2						30.0800
	Sig.		1.000	.080	.087	1.000	1.000	1.000

Means for groups in homogeneous subsets are displayed.

a. Uses Harmonic Mean Sample Size = 2.000.
